# Pigs solve a cooperative task without showing a clear understanding of the need for a partner

**DOI:** 10.1038/s41598-024-84529-3

**Published:** 2025-02-11

**Authors:** Jim McGetrick, Kimberly Brosche, Clémence Nanchen, Jean-Loup Rault

**Affiliations:** 1 Institute of Animal Welfare Science, University of Veterinary Medicine, Vienna, Veterinärplatz 1, 1210 Vienna, Austria; 2 Department of Behavioral & Cognitive Biology, University of Vienna, Djerassiplatz 1, 1030 Vienna, Austria

**Keywords:** Coordination, Joint action, Porcine, Social cognition, Social behaviour, Animal behaviour, Social evolution

## Abstract

Many animal species engage in cooperation, whereby they act together, typically to achieve a common goal. Domestic pigs were recently shown to lift a log together in pairs in the joint log-lift (JLL) task to access food treats. However, it is not yet clear whether pigs understand that they need a partner for this task. To investigate this, we applied a recruitment approach similar to that used for chimpanzees, coral trout, wolves and dogs. Pigs that were proficient with the JLL task were given access to the task on their own but could open a door to recruit a familiar partner from an adjacent enclosure. Pigs opened the door in all experimental conditions, allowing the partner to enter (if a partner was present). Comparing across conditions, latencies to open the door and to return to the box after opening the door generally did not suggest that subjects understood the need for the partner. As with many animal species in cooperative tasks, pigs may achieve a complex cooperative outcome in the JLL task without a full understanding of the need for a partner.

## Introduction

Cooperation has been defined as the simultaneous or consecutive acting together of two or more individuals by the same or different behaviours^1^. Cooperation can be observed in non-human animals in a variety of contexts including cooperative hunting or foraging (e.g. chimpanzees^2–4^ [*Pan troglodytes*], wolves^5^ [*Canis lupus*], lions^6,7^ [*Panthera leo*], African wild dogs^8^ [*Lycaon pictus*], killer whales^9,10^ [*Orcinus orca*], humpback whales^11,12^ [*Megaptera novaeangliae*], bottlenose dolphins^13^ [*Tursiops truncatus*], Galapagos sea lions^14^ [*Zalophus wollebaeki*], and Harris’ hawks^15^ [*Parabuteo unicinctus*]); anti-predator behaviour (e.g. pronghorn antelope^16^ [*Antilocapra americana*], bighorn sheep^17^ [*Ovis canadensis*], pied flycatchers^18^ [*Ficedula hypoleuca*], and bluegill fish^19^ [*Lepomis macrochirus*]); and cooperative transport (e.g. ants^20^ [*Hymenoptera: Formicidae*]). However, observational studies such as these are typically insufficient to draw conclusions regarding the cognition involved in cooperation. The lack of clarity surrounding the cognition involved in cooperative interactions in nature has resulted in a spate of studies applying experimental cooperation tasks which allow for fine-grained manipulation of cooperative situations and subsequent elucidation of underlying proximate mechanisms (see Noë^21^; McAuliffe and Thornton^22^, Albiach-Serrano^23^, and Duguid and Melis^24^ for review).

The question of whether non-human animals understand the need for the partner in cooperative interactions has been addressed in many experimental studies with a variety of species (see Albiach-Serrano^23^ and Duguid and Melis^24^ for review). One of the most common approaches to testing for such understanding has been the so-called “delay condition” (or “delay task”) which has been applied in the loose-string paradigm^25–38^. In the loose-string paradigm, a string or rope is threaded through a baited platform (or apparatus) outside the reach of study participants and requires two individuals to pull the ends of the rope simultaneously to bring the food into reach; one individual pulling only at one end results in the string/rope becoming unthreaded^38^. In the delay condition, a subject is typically given access to the task on its own and a partner is released after some delay. If the subject fails to wait for the partner and attempts to pull alone, it unthreads the string/rope, thereby disabling the task, and it does not gain access to the food. To demonstrate an understanding of the need for a partner in this task, the subject must refrain from pulling the string/rope until the partner arrives and pulls.

Some indoor-housed farm pigs^39^ (*Sus scrofa domesticus*) and free-range Kune Kune pigs^40^ were recently shown to effectively cooperate in a similar task – the joint log-lift (JLL). The JLL task consists of a box containing baited food bowls behind a horizontally set wooden log. The food inside the box can only be accessed if pairs of individuals simultaneously lift the log upwards, out of the way. This task is analogous to cooperative pulling^38,41,42^ and cooperative stone-moving^43^ tasks designed for primates in that it cannot be solved by one individual acting alone. However, as pigs’ natural behavioural repertoire includes rooting^44,45^ and lifting^46^ with their snouts, rather than pulling, the JLL makes for a more species-appropriate task. However, it is not yet clear whether pigs understand that a partner is required for success.

To probe into their understanding of the need for a partner, Kune Kune pigs were tested with a delay condition in the JLL task, experiencing two different delay durations (10 s and 30 s^40^). Subjects did not wait for their delayed partner in these periods and proceeded to interact with the apparatus before the partner was released, apparently attempting to lift the log alone. However, as highlighted by Koglmüller et al.^40^, unlike the loose-string task in which attempts in the absence of a partner are detrimental, attempts to lift the log in the JLL task in the absence of a partner do not disable the mechanism. Thus, there is little incentive to refrain from lifting the log until the partner has arrived. Failure to wait for the partner in a delay condition is not, therefore, a reliable indicator of pigs’ lack of understanding of the need for a partner in this particular task. Furthermore, the pigs in this study had to be trained initially to lift the log individually, which may have impaired their understanding of the later cooperative nature of the task.

An alternative for investigating understanding of the need for a partner is the recruitment approach. Rather than releasing the subject first and releasing the partner after some delay, the subject can be presented with the cooperative task alone and given the option of actively recruiting a partner. Melis et al.^47^ provided chimpanzees with this option in a loose-string task. After having learned how to successfully complete the task with a partner, and after passing a delay condition and learning how to unlock a door separating two enclosures, individual subjects were presented with the task on their own. Subjects recruited a partner from the neighbouring enclosure by unlocking the door significantly more often when they needed the partner than in a control condition in which the two ends of the rope were close enough together for the subject to pull and succeed alone. Similarly, coral trout^48^ (*Plectropomus leopardus*), wolves and domestic dogs^37^ (*Canis familiaris*) have since been shown to recruit a partner when needed in a cooperative task.

In the current study, we investigated whether pigs understand that they need a partner to succeed in the JLL task, using a similar recruitment approach. After learning how to successfully lift the log with a partner in the JLL task, and how to lift logs individually in a control, “individual log-lift” (ILL) task, subjects were given the opportunity to learn how to open a small door between two enclosures. Later, in test sessions, whether a subject needed a partner, and whether a partner was available, was varied across one test condition and four control conditions. If pigs understand that they need a partner to succeed in the JLL task, we predicted that their latency to open the door and their latency to return to the box after opening the door would be shorter when a partner was both needed and present, than in all other experimental conditions. In addition, we expected that pigs that were more proficient in our spontaneous learning phase may understand the task better (i.e. would differentiate more strongly between the conditions).

## Methods

### Ethical approval

All procedures were approved by the animal ethics and animal welfare committee of the University of Veterinary Medicine, Vienna (protocol numbers: ETK-044/03/2021; ETK-093/06/2021), in accordance with the Good Scientific Practices guidelines and national legislation, and authors complied with the ARRIVE guidelines. If pigs showed signs of wanting to escape during an experimental session, the test session was terminated. This occurred only once.

### Subjects and housing

This study was conducted on a teaching and research pig farm, owned and managed by the University of Veterinary Medicine, Vienna. Thirty-six pigs (Large White × Pietrain breeds; 18 females and 18 castrated males) were included in the study. These pigs were selected initially from at least six different litters and were approximately 4 weeks of age at the beginning of the study. The pigs were divided into six groups, each composed of three females and three males, with no littermates in the same group. Mixing of non-littermates after weaning is a common farm practice. Although this can lead to aggression while the pigs establish their hierarchy, these agonistic interactions usually vanish after 3 days^49^, which was long before the testing phase in this study.

The groups were housed in partly slatted-floor pens (average dimensions: 432 cm × 244 cm) with a covered, heated sleeping area (average dimensions: 244 cm × 100 cm; cover 94 cm above ground). They had *ad libitum* access to commercial pig meal through a multi-space feeder and *ad libitum* access to water through a drinking trough. Each group was provided with environmental enrichment in the form of wood shavings, straw, a small orange ball (Dogs Creek Ball Airflow M-L; diameter, 7.6 cm; cat no. 1337241; MULTIFIT Tiernahrungs GmbH, 47809 Krefeld, Germany) and two jute ropes (thickness: 20 mm) which were hanging in the enclosure. For identification purposes, throughout the study each pig received a mark on its back approximately once a week with a livestock marker spray. The study lasted for six weeks during the summertime (July – August) with all aspects of exposure, learning phases and testing being carried out on consecutive week days.

### Apparatuses

#### Joint log-lift box

**Fig. 1:**
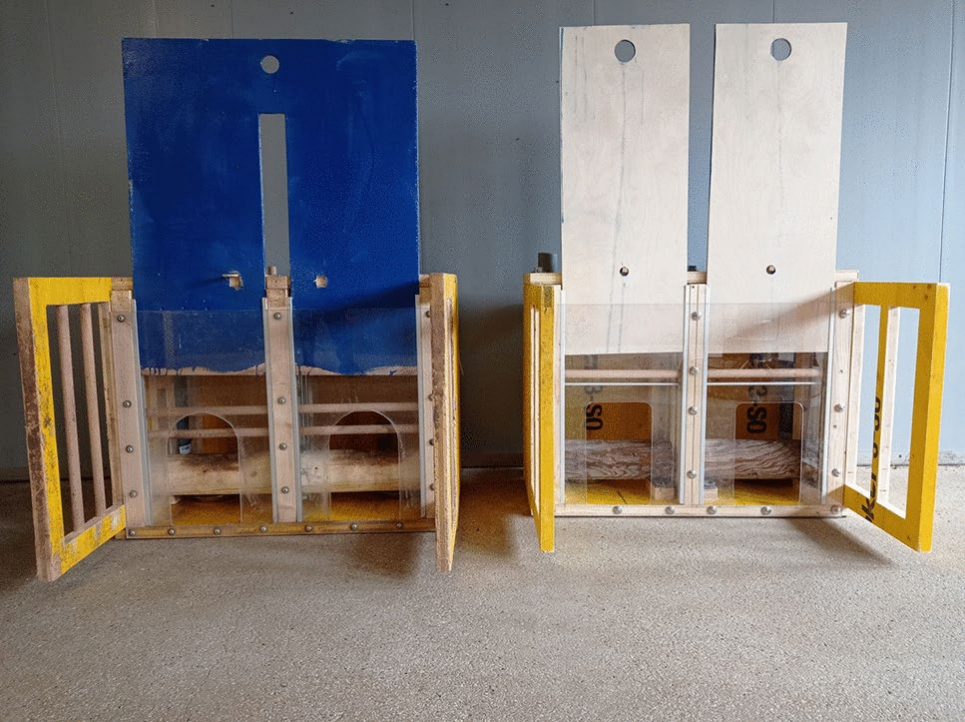
Left, joint log-lift (JLL) box with front panel open and log fully down. Right, individual log-lift (ILL) box with both front panels open and both logs fully down.

The joint log-lift (JLL) box (57.2 cm × 75.4 cm × 43.7 cm) was a modified version of that used by Rault et al.^39^. It contained a horizontally resting log (length = 67.5 cm; diameter = 10 cm) that needed to be lifted by two pigs simultaneously to access food treats inside (see Fig. 1). Once the log was lifted to a height of approximately 14.5 cm, the log was locked in place by a spring-loaded bolt latch until the experimenter released it again. The front of the box was fitted with a transparent acrylic sheet with two openings, one to the left and one to the right, allowing the pigs to insert their snouts into the box to lift the log and to insert their heads to access food treats. A sheet of plywood directly behind the acrylic sheet could be raised and locked in the upper position to allow the pigs access to the log, or lowered to block access to the log. An experimenter could bait the box by dropping food treats down through two pipes leading to bowls within the box. Fences (~ 29 cm in length) protruded from either side of the box to allow two pigs access to the log without being displaced by others from the flanks. A dowel was inserted in each box horizontally, directly behind the acrylic sheet and plywood, to prevent the pigs from walking over the log to access the food when the log was fully down. The positioning of the dowel could be raised as the pigs grew. Successful lifting of the log, such that it locked into its highest position, required two pigs to synchronize their lifting i.e. to lift the log at the same time with their snouts. For diagrams of the JLL box, see Supplementary material ESM_1, Supplementary Fig. 1.

#### Individual log-lift box

The individual log-lift (ILL) box was similar to the joint-log lift box, with some notable differences, as outlined here (see Fig. 1). Rather than a single long log that required two pigs to work together to lift, it consisted of two short logs (length = 30.5 cm; diameter = 10 cm), one to the left and one to the right. These two logs could move independently of each other, thereby allowing two pigs to work side-by-side, but independently, to obtain food treats. Directly behind the acrylic sheet at the front of the box were two plywood panels, one to the left and one to the right such that access to each log could be provided or blocked independently of the other. For further details, see the supplementary methods.

For both boxes, one face of the plywood sheet(s) was painted blue and one face remained unpainted. This meant that the front face of each box could appear blue or not depending on which way around the plywood sheets were inserted. Half of the six groups of pigs experienced the JLL with a blue front face and the ILL with an unpainted front face, whereas the other half of the pigs experienced the JLL with an unpainted front face and the ILL with a blue face. The differently coloured box front faces for each group were included to assist in the pigs’ recognition of the two box types. Behavioural^50^ and physiological evidence^51^ suggests that pigs are able to perceive blue and should be able to dinstinguish it from the colour of the unpainted plywood.

### Log-lift box exposure and learning phase

#### Experimenters

Two female experimenters conducted the log-lift box learning phase, wearing identical clothing. Throughout the learning phase, one experimenter conducted all sessions with the JLL and the other conducted all sessions with the ILL.

#### Provision of food treats

For the first two days of the study, each group of pigs was provided with pieces of apple, in order to familiarize them with this food type, used throughout the study. An experimenter entered the enclosure, with a bowl/handful of apple pieces (each ~ 1 cm^3^) and handed pieces to each pig individually for approximately 15 min per group.

#### Exposure to the boxes

On the subsequent three days, the pigs were exposed to the two boxes separately in their home enclosure. Each box was placed inside the enclosure with the wooden panel closed and the log(s) locked in the upper position, so that food treats could be accessed in order for the pigs to learn the location of the food treat. Apple pieces were dropped through the pipes into the food bowls and the panel(s) was (were) then lifted. The pigs were given 15 min to inspect and interact with each box and to eat the apple pieces in the food bowls. Apple pieces were dropped down through the pipes frequently throughout the session to ensure that there was always food available. At the end of the 15-min session, the wooden panel(s) was (were) closed and the box was removed from the enclosure. The order in which the two boxes were presented to each group was varied systematically across days. In addition, the order in which the different groups were presented with the boxes was varied systematically across days. Due to experimenter error, the colour of the front panel of the two boxes did not differ within groups on the first day.

#### Learning to lift the log

The log-lift learning phase took place over 12 days and was similar for the two boxes. Each group had a 15-min session with each box type on each of these days. The box was brought into the pigs’ enclosure at the beginning of the session, with the front panel closed. When the box was positioned appropriately, the experimenter dropped approximately three apple pieces down each pipe, into the bowls. The experimenter then began a 15-min timer and lifted the front panel(s), revealing the log(s) in the lowest possible position. The order in which the boxes were presented to each group and in which the different groups were presented with the boxes was varied across days in a systematic manner.

In the case of the JLL box, if the pigs succeeded in lifting the log together such that it locked in the upper position, they could retrieve apple pieces inside the bowls. Once the pigs obtained these food treats and retreated from inside the box, the experimenter closed the front panel, thereby blocking access to the inside of the box, released the log, such that it dropped back to its lowest position, rebaited the bowls, and lifted the front panel again.

The procedure with the ILL box was similar; however, given that each log could be lifted and locked in the upper position individually, and that the two front panels could be moved individually, operation of the two sides of the ILL box was not synchronized (e.g. at any given moment, the log on one side of the box may have been accessible while the experimenter may have been in the process of resetting the log on the other side, with the front panel on that side closed).

The frequency of successful lifts of the logs (with a successful lift defined as locking of the log in the upper position) over this 12-day learning period was recorded live by the experimenters. The change in performance across the first 10 days is presented in the supplementary material (see Supplementary material ESM_1; Supplementary Table 1 and Supplementary Fig. 2). The identities of the successful individuals with the JLL, for later selection of dyads for the test sessions, was determined using video footage. Despite the design of the box, due to their developing strength, on the last day of this learning phase, four subjects managed to lift the log in the JLL alone (one subject lifted alone once, the other three lifted alone twice each). Given that this occurred a minimal number of times, these pigs were nonetheless included in the test sessions and subsequent analysis.

### Test enclosure and door opening

**Fig. 2:**
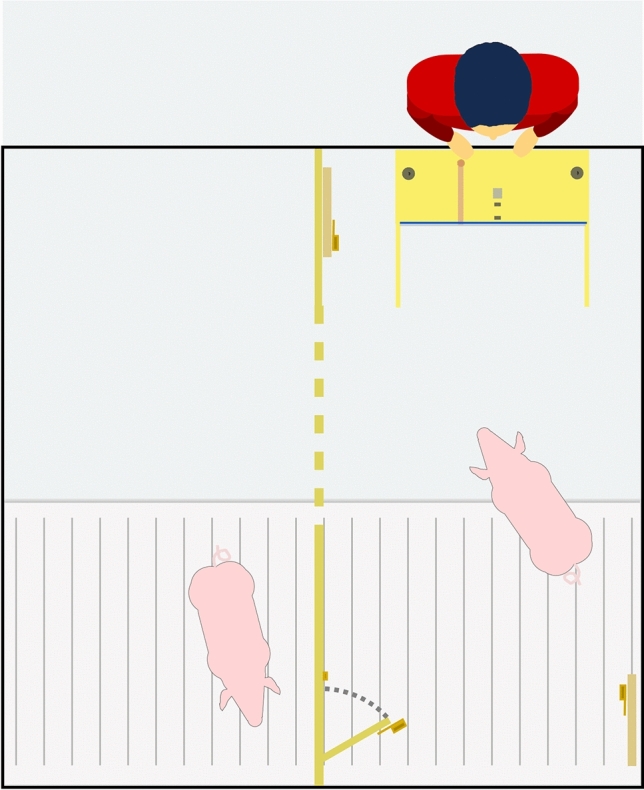
Overview of test enclosure setup during a test session. The box was placed against the back wall of the subject’s compartment (right). A wooden wall separated the subject’s compartment from the partner’s compartment (left). A small door, which could be opened from the subject’s compartment, allowed the partner to enter the subject’s compartment, if opened. Two decoy handles were mounted in the subject’s compartment on panels of wood, one to the left (on the separating wall) and one to the right.

The test enclosure (i.e. the enclosure to be used in the final experimental sessions; 244 cm × 250 cm) was similar in layout to the home pen (see Fig. 2). It was divided into two equal compartments (approx. 122 × 250 cm), the subject’s compartment and the partner’s compartment, by a wooden wall (250 cm × 100 cm; see Supplementary material ESM_1, Supplementary Fig. 3). The wall contained a small door (30 cm × 30 cm) at one end that could be opened from one compartment only (the subject’s compartment) by lifting a latch (positioned approx. 6 cm from the ground)^52^. The door was large enough for one pig to pass through at a time. The wall also contained multiple narrower openings that allowed pigs to see what was in the adjacent compartment. Two decoy door latches were also present in the test enclosure, to allow for assessment of the pigs’ motivation to interact with the door latches, if necessary. These latches were identical to the latch that could be used to open the door, and they could be lifted, but they were not attached to a door that could be opened. One latch was attached to the wall directly opposite the functional door (referred to as “right handle”) while the other was positioned on the dividing wall at the opposite end to the functional door (referred to as “left handle”). Analysis of the proabiity of interacting with these three different door handles in the test sessions is included in the Supplementary material ESM_1.

To accustom the pigs to the test enclosure and to allow them to learn how to open the door, the pigs were given 15-min sessions in the test enclosure in groups of three for five days. The three pigs were brought to the subject’s compartment and were allowed to explore it. On the first two days, the small door was open at the beginning of the session such that the pigs were able to freely move from the subject’s compartment to the partner’s compartment and back again. On subsequent days, the small door was closed at the beginning of the session. The pigs could lift the latch to open the door and then explore the partner’s compartment. After 2 min of exploring the partner’s compartment, at least one pig was moved back to the subject’s compartment and the door was closed again. This procedure was carried out to ensure the pigs had more than one opportunity to open the door in each session. The pigs sometimes closed the door themselves, resulting in additional opportunities to open the door. If one pig monopolized the door handle, the other pigs were more frequently directed back to the subject’s compartment to provide more opportunity for these pigs to open the door. If the pigs failed to open the door after 10 minutes, an experimenter opened the door. To be considered suitable for participation as a subject in the recruitment test, pigs were required to have opened the door at least three times in total across the five sessions. The groups of pigs brought to the test enclosure for these sessions were pen mates. The composition of this group of three varied from day-to-day as the group members were randomly selected from the larger group of six each time, with the exception that the selected pigs had to have already been successful in lifting the log with a partner. The maximum of door openings performed by an individual across this entire learning phase was 28 and the minimum was six. The number of door openings significantly increased across the five days (see Supplementary material ESM_1; Supplementary Table 2 and Supplementary Fig. 4).

### Formation of dyads for the test

Fifteen dyads were selected to participate in the test sessions (see Supplementary material ESM_1, Supplementary Table 3). The selection of dyads was based on an attempt to maximize sample size without testing the same individuals in multiple dyads. All individuals selected to participate in the test sessions had lifted the log in the JLL task at least 17 times during the learning phase (i.e. with any partner) with the majority of individuals having lifted the log a considerably greater number of times (range: 17–424; median: 132; mean ± S.D.: 146.76 ± 108.87). These values are based on successes by the eleventh day of the log-lift learning phase. Within the dyads selected for the test sessions, the frequency of successful lifts of the log together in JLL task during the initial learning phase ranged from one to 86, again based on figures after day eleven of the learning phase. In one of these dyads, only one individual was tested as a subject; the partner had not been given the opportunity to learn how to open the door due to not having succeeded with the JLL prior to selecting pigs for the door opening learning phase.

### Test procedure

Test sessions took place over five consecutive days and consisted of five experimental conditions (joint log-lift, asocial, individual log-lift, log up – empty, log up – full; see “conditions” section below). The procedure across test conditions was similar. Prior to the test session, one of the boxes was brought into the subject’s compartment of the test enclosure and set up against the back wall, with the front panel(s) of the box closed. The choice of box, whether the food bowls inside the box were baited, the position of the log, and whether a partner was present in the adjacent enclosure, all depended on the experimental condition. Directly before each experimental session, the dyad was brought to the test enclosure. They were directed into the partner’s enclosure and remained there together for 1 min to allow them to settle. In one of the experimental conditions (see below), no partner was present; thus, the subject was brought to the partner’s compartment of the test enclosure alone. After 1 min had elapsed, the small door between the compartments was opened and the subject was directed through this door into the subject’s compartment. The small door was closed again immediately and all three door handles in the subject’s compartment were covered by wooden covers attached to long wooden handles which were held by the experimenters from outside the enclosure. The subject remained in this compartment, with the door handles covered and the front panel of the box closed, for 30 s to allow a settling period before the session began. After 30 s had elapsed, the front panel(s) of the box was (were) lifted and the covers on the door handles were removed. The subject was then given a 5-min testing period. We provided the pigs with a 5-min testing period based on our subjective assessment of how much time would be sufficient to open the door and lift the log. If a log was lifted in this session, it was not reset. Similarly, if the small door was opened by the pig, it was not closed again by an experimenter. During the 5-min test session, one experimenter stood outside the enclosure behind the box and was visible to the pigs. The second experimenter stood approximately 2–3 m away from the enclosure and was not visible to the pigs. After this 5-min test session had elapsed, the front panel of the box was closed and both pigs were removed from the test enclosure. The roles of the two experimenters remained consistent throughout the entire testing period. An example of the procedure can be seen in the Supplementary video ESM_2.

Each pig experienced two sessions per day, once as a subject and once as a partner, as the roles (within the dyad) were reversed after the first session. There was a 15-min break in between these two sessions and the condition that a subject experienced on a given day was the same as the condition that their partner later experienced as a subject. The order in which pigs were tested each day, and the exact time of day that a particular subject was tested, remained consistent across the five days. The order in which conditions were experienced over the five days was randomized across dyads. The colour of the front panels of each box matched that which a dyad had experienced during the learning phase. In one experimental session on the first test day, the partner managed to lift the log alone. To prevent this occurring in future experimental sessions, the experimenter held a block of wood above the opening at the top of the box, thereby blocking the movement mechanism of the log, until the subject and partner were present at the box together; this ensured that if one individual attempted to lift the log alone, it could not be lifted high enough to lock in the upper position.

### Conditions

Five conditions were tested in this study, as follows:

#### JLL condition

The JLL condition was the main test condition. Here, the JLL box was present in the subject’s compartment of the test enclosure. At the beginning of the test session, the log was in its lowest position, the food bowls were baited (with approximately four apple pieces per bowl), and there was a partner present in the partner’s enclosure. To successfully lift the log, the subject needed to open the small door to allow the partner into the subject’s compartment to help. An example of the JLL condition can be seen in the Supplementary video ESM_2.

#### Asocial condition

The asocial condition was identical to the JLL condition with the exception that no partner was present. This meant that it was not possible for the subject to succeed in lifting the log, regardless of whether it opened the small door or not. This condition was included to allow us to determine whether the subjects’ decision to open the small door was influenced by the presence of a partner.

#### ILL condition

The ILL condition was identical to the JLL condition but instead of the JLL box, the ILL box was present in the subject’s compartment. The logs were in their lowest position and the bowls were baited at the beginning of the session. Given that this was a non-cooperative task for which a partner was not needed, the inclusion of this condition allowed us to determine whether the pigs’ decision making during the test was dependent on their need for a partner. An example of the ILL condition can be seen in the Supplementary video ESM_2.

#### Log up – empty

The log up – empty (LUE) condition was identical to the JLL condition with the exception that the log was fixed in its highest position from the beginning of the session and no food was present in the box. In this condition, there was, therefore, no task to solve and no food to be acquired. This condition was included as an additional condition to determine whether pigs’ decision making was dependent on their need for a partner, but in the presence of the JLL box. The colour of the front panel matched the colour the dyad was used to with the JLL box.

#### Log up – full

The log up – full (LUF) condition was identical to the LUE condition with the exception that there was food present in the bowls. As with the LUE condition, there was no task to solve. The bowls were baited in this condition to allow us to rule out the acquisition and consumption of food as an explanation for any differences observed between the JLL and ILL conditions.

#### Predictions and expectations

We expected the subjects to open the small door in all conditions, as five minutes was a relatively long time for them to explore the compartment and manipulate objects within. However, if the pigs understood that they needed a partner to help them with the JLL task, we predicted that their latency to open the door in the JLL condition would be shorter than in all other conditions as it was the only condition in which a partner was not only needed but also available.

It was possible that pigs needed to inspect the box, or even attempt to lift the log, before deciding whether a partner was needed. In the ILL condition, this could have resulted in lifting one or both logs and obtaining food. Similarly, in the LUF condition, inspection of the box could have resulted in the pigs obtaining food. Thus, the latency to open the small door in these conditions may have been longer than in the JLL condition, not because the subjects were deciding against recruiting a partner, but rather because they were pre-occupied with lifting logs or recovering the food. Thus, to account for this potential distraction, we decided to also analyse the latency to open the door from the last moment the subjects were in proximity to the box prior to the door being opened. If subjects understood the need for a partner, we predicted that this latency in the JLL condition would be shorter than in all other conditions.

It was also possible that subjects would open the door *before* reaching the box in some sessions, meaning that a latency to open the door *after* the last moment they were in proximity to the box would not exist for these sessions. To determine whether the presence/absence of this latency was significantly biased towards some conditions, we analysed the probability of opening the door before reaching the box. This analysis also provided the opportunity to determine whether they responded differently to the two different box types (or to the different conditions) in terms of their decision to open the door before reaching the box, or vice versa, potentially providing an insight into whether they discriminated between the boxes or not. For example, if subjects understood the need for a partner, they may have been more likely to open the door before reaching the box in the JLL condition than in all other conditions (note, however, that subjects could have been in proximity to the box from the beginning of the session).

We also predicted that the subjects’ latency to reach the box after opening the small door would be shorter in the JLL condition than all other conditions, as individuals that open the door to recruit a partner should be more motivated to return to the box in this condition.

Given that we allowed the pigs to learn log-lifting and door-opening spontaneously, without providing specific training sessions, variation in log-lifting and door-opening proficiency (i.e. the number of times they lifted the log successfully or opened the door successfully) emerged among the study participants. We expected that this may influence their likelihood of understanding the task or performing well in the test sessions. Therefore, we took this variation in proficiency into account in our analysis.

### Behaviour coding

Coding of behaviours was carried out by one experimenter using Solomon Coder (version beta 19.08.02^53^). The following behaviours were coded as instantaneous events for the subject: first proximity to the box before the door was opened, last proximity to the box before the door was opened, door opening, first proximity to the box after the door was opened, and success in lifting the log. First proximity to the box before the door was opened was defined as the first time the subject’s head entered the rectangular zone between the two fences protruding from the apparatus (this was only coded if it happened before the door was opened; also, subjects could have already been in proximity to the box when the session began). Last proximity to the box before the door was opened was defined as the last frame in which the subject’s head was within the zone between the two fences before it opened the door. Door opening was defined as the first frame in which the door was visibly ajar after the subject had lifted the latch. First proximity to the box after the door was opened was defined as the first frame in which the subject’s head entered the zone between the two fences protruding from the apparatus after the subject had opened the door. Success in lifting a/the log was defined as the log locking in the highest position, accompanied by a visible signal/hand gesture the experimenter gave to the camera. A second person coded 20% of the experimental sessions for interobserver reliability analysis. The number of times the small door was opened during the door-opening learning phase was scored manually and the probability of interacting with each of the three door handles during the test sessions was coded using Solomon Coder.

For analysis, we extracted the latency for the subject to open the door from the beginning of the test session, the latency to open the door following the last moment the subject qualified as being in proximity to the box (before the door was opened), and the latency to reach proximity to the box after the door was opened. Note that if subjects opened the door before reaching proximity to the box, they did not have a latency to open the door following the last moment they were in proximity to the box (this occurred in 57 out of 203 trials).

### Week 2

Over the course of the testing sessions, prior to having coded the videos or having analysed the data, based on subjective assessment of performance in the test, we deemed it plausible that the pigs were not particularly focused on the presence of the box in the test enclosure, as they were not accustomed to encountering it there. Prior to the test, they had only ever experienced the boxes in their home enclosure. Therefore, we decided to provide learning sessions with the boxes in the test enclosure before testing the subjects in a second round. Given the limit on the duration of availability of the pigs, there was only time to test two experimental conditions in a second week of testing. We tested the pigs with the JLL and the ILL conditions in this second week after additional exposure to the boxes in the test enclosure.

### Exposure to boxes in the test enclosure (week 2)

The exposure to the boxes in the test enclosure took place over three days after the first week of testing was complete. One of the boxes was placed in the subject’s compartment of the test enclosure with the log(s) down, the bowls baited and the front panel(s) closed. The pig dyad (the same pairing as used in the test sessions) was brought into the partner’s compartment of the test enclosure and was allowed to settle there for 1 min before the small door between the compartments was opened by an experimenter. The pigs were both directed through the door into the subject’s compartment and the door was closed behind them. All door handles that were present were then covered for the duration of the session. Once one of the experimenters was in place outside the enclosure to operate the box, the front panel(s) of the box was (were) lifted and the pigs were free to interact with the log(s) for 5 min. Throughout this 5-min session, the log(s) was (were) reset and the bowls were rebaited exactly as was carried out during initial learning sessions in the pigs’ home enclosure.

After 5 min had elapsed, the experimenter closed the front panel(s) and the second experimenter removed the cover from the handle of the small door. The pigs were free to open the small door once. Typically, if the door was not opened by one of the pigs within approximately 2 min of the session ending, an experimenter opened the door. Once the door was opened, the pigs were directed back into the partner’s enclosure and the small door was closed behind them. The pigs remained in the partner’s compartment while the box in the subject’s compartment was exchanged for the alternative box (i.e. if the JLL box had been in the subject’s compartment, it was replaced with the ILL box). When the appropriate box was in place in the subject’s compartment (usually after approx. 1 min), the small door was opened again. The procedure for the second box resembled that described for the first box. When the 5-min session ended, the front panel(s) was (were) closed and the pigs were permitted to open the small door and then they were returned to their home enclosure. The order in which pigs experienced the two box types was alternated across dyads and days.

In contrast with the first learning phase, only one experimenter conducted most learning sessions with both boxes. The acrylic sheet on the front of each box was removed during this learning phase (on the first day after the sessions with the first four dyads) as some pigs had grown too big to fit through the openings to reach the food. The two decoy door handles were also removed on the first day after the sessions with the first four dyads, to avoid distraction in future sessions. Dyads were identical to those used in the initial round of testing. One of the dyads did not take part in a session on the first day due to experimenter error; however, they did take part on the second and third days.

### Test procedure (week 2)

The test sessions took place over the two days following the learning phase. The test sessions were performed exactly as described for the first week of testing, and at the same time of day as the first week for each dyad. Half of the dyads experienced the JLL condition on the first day and the ILL condition on the second day. The other half of the dyads experienced these two conditions in the opposite order. Prior to these test sessions, an extra space (height = 4 cm) was cut out above the small door to increase its height as some of the pigs had grown too big to comfortably fit through since the previous test sessions.

### Behaviour coding (week 2)

Behaviour coding for the second testing was carried out in an identical manner to that for the first testing.

### Statistical analysis (week 1)

All models were fitted in R (version 4.0.2—4.3.0^54^). The packages and functions used in each case are given below. Random slopes were identified, and confidence intervals were obtained for plotting, using functions kindly provided by Roger Mundry (current affiliation: German Primate Center – Leibniz Institute for Primate Research, Göttingen, Germany). Cumulative incidence plots were created using the package “survminer” (version 0.4.7^55^). Interobserver reliability for latencies was assessed by calculating the intraclass correlation coefficient using the function “icc” in the package “irr” (version 0.84.1^56^), setting the “model” argument to “twoway” and the “type” argument to “consistency”. Interobserver reliability regarding whether the behaviour of interest occurred or not was assessed by computing Fleiss’ Kappa using the “kappam.fleiss” function in the package “irr” (version 0.84.1^56^). The behaviours for which Fleiss’ Kappa was evaluated included whether the door was opened after reaching proximity to the box, whether the box was reached after opening the door, and whether the subject was successful in lifting the log.

#### Probability of opening the door before reaching the box

To determine whether the condition influenced the pigs’ decision to open the door before reaching the box (or vice versa), we analysed the probability of opening the door before reaching the box. We fitted a generalized linear mixed model (GLMM) with a binomial error distribution and a logit link function^57^ (0, reached the box first; 1, opened the door first). We included condition as the main predictor of interest. To control for the differing levels of competence and experience of the subjects with regards to log lifting and door opening, as fixed effects we also included the number of times the subject had lifted the JLL successfully with any partner in the learning phase and the number of times the subject had opened the door successfully during the learning phase. A two-way interaction was included between condition and both of these fixed effects. Condition order (i.e. the order in which conditions were experienced) was included as an additional fixed effect. We included random intercept effects of subject, dyad (i.e. the subject-partner pairing), and group (i.e. the group that the subject was housed in throughout the study period). To avoid overconfidence regarding precision of the estimates for the fixed effects, and to ensure type *I* error rate remained at the nominal level of 5%^58,59^, condition and the number of times the JLL was lifted during the learning phase were included as random slopes within the random effects of dyad and group. Condition was manually dummy coded and centred for inclusion as a random slope. We z-transformed numerical variables to a mean of zero and a standard deviation of one prior to fitting the model to facilitate interpretation of results and to ease model convergence. We fitted the model using the function “glmer” from the package “lme4” (version 1.1–33^60^). Correlations among the random intercepts and random slopes were removed.

As an overall test of the effect of condition and its interactions, we conducted a full-null model comparison^61^ to avoid cryptic multiple testing. The null model lacked the fixed effect “condition” and its interactions but was otherwise identical to the full model. This comparison was based on a likelihood ratio test^62^ using the function “anova” and setting the “test” argument to “Chisq”. The sample for this model included a total of 144 observations across 29 subjects, 15 dyads, and 6 groups.

#### Latency to open the door

To analyze the effect of condition on the latency to open the door from the beginning of the session, we fitted a Cox proportional hazards mixed effects regression model. The response variable included the latency to open the door and whether the event of opening the door occurred. This was entered using the “Surv” function in the package “coxme” (version 2.2–18.1^63^).

The fixed effects and random intercepts were identical to the previous model. No random slopes were included. We fitted the model using the function “coxme” in the package “coxme” (version 2.2–18.1^63^). Prior to fitting the model, we z-transformed numerical variables. As above, we conducted a full-null model comparison to test for the effect of condition and its interactions. The sample for this model included a total of 144 observations across 29 subjects, 15 dyads, and 6 groups.

Interobserver reliability was excellent (ICC = 1, n_observations_ = 29 [1 NA excluded], n_raters_ = 2, p < 0.001; Kappa = 0.78, n_observations_ = 29, n_raters_ = 2, p < 0.001). Post-hoc pairwise comparisons of the conditions were carried out using the function “emmeans” in the package “emmeans” (version 1.8.6^64^), setting the “specs” argument to “pairwise ~ condition”.

#### Latency to open the door following last proximity to the box

To analyze the effect of condition on the latency to open the door following the last moment the subject was in proximity to the box, we fitted a Cox proportional hazards mixed effects regression model using the same method described above for “latency to open the door” but replacing the response variable. The sample for this model included a total of 100 observations across 29 subjects, 15 dyads, and 6 groups. The number of observations for this analysis was smaller than for the previous model, as some subjects opened the door before reaching proximity to the box and, therefore, did not have a latency for the current variable.

Interobserver reliability was excellent (ICC = 0.908, n_observations_ = 29 [13 NAs excluded], n_raters_ = 2, p < 0.001; Kappa = 0.784, n_observations_ = 29, n_raters_ = 2, p < 0.001).

#### Latency to reach proximity to the box after opening the door

The effect of condition on the latency for the subject to reach proximity to the box after opening the door was analysed using the same approach as described above but with the appropriate response variable included. The sample for this model included a total of 137 observations across 29 subjects, 15 dyads, and 6 groups.

Interobserver reliability was excellent (ICC = 0.96, n_observations_ = 29 [3 NAs excluded], n_raters_ = 2, p < 0.001; Kappa = 0.79, n_observations_ = 29, n_raters_ = 2, p < 0.001).

### Statistical analysis (week 2)

All variables were analysed in the same manner as week 1 with some changes highlighted below. For the model analysing the probability of opening the door before reaching the box, condition and the number of times the JLL was lifted during the learning phase were included as random slopes within the random effects of dyad and group. The sample for this model included a total of 58 observations across 29 subjects, 15 dyads, and 6 groups. Analysis of latency to open the door included a total of 58 observations across 29 subjects, 15 dyads, and 6 groups. Analysis of latency to open the door following last proximity to the box included a total of 45 observations across 28 subjects, 15 dyads, and 6 groups. Analysis of latency to reach proximity to the box after opening the door included a total of 51 observations across 28 subjects, 15 dyads, and 6 groups.

#### Latency to succeed with the JLL in week 1 vs week 2

Given that the general pattern of results for week 2 was similar to week 1, it was unclear whether the additional exposure to the test setup before testing in week 2 had any impact on performance. We decided to compare the latencies to succeed in the JLL in week 1 and week 2, as we expected to see some improvement with regards to this variable following the additional exposure to the test setup.

The effect of week (i.e. week 1 vs week 2) on the latency to successfully lift the log with a partner in the JLL condition was analysed using a Cox proportional hazards mixed effects regression model as above.

We included week as a fixed effect and subject, dyad, and group as random effects. We included the random slope of week within the random effects of dyad and group. Prior to insertion as a random slope, the factor week was dummy coded and centred.

The correlation between the random slope of week and the random intercept of group was estimated to be very close to minus one and was removed with only a minor reduction in model fit (log-likelihoods: model with correlation: -53.71 [df = 6.69]; model without correlation: -54.09 [df = 7.50]). The null model lacked the fixed effect of week but was otherwise identical to the full model. The sample for this model included a total of 58 observations across 29 subjects, 15 dyads, and 6 groups.

Interobserver reliability was excellent (ICC = 1, n_observations_ = 29 [23 NAs excluded], n_raters_ = 2, p < 0.001; Kappa = 0.901, n_observations_ = 29, n_raters_ = 2, p < 0.001).

## Results

### Week 1

#### Probability of opening the door before reaching the box

The full-null model comparison revealed no effect of condition or its interactions on the probability of opening the door before reaching proximity to the box (full-null model comparison: χ^2^ = 11.158, *df* = 12, *P* = 0.515).

#### Latency to open the door

**Fig. 3:**
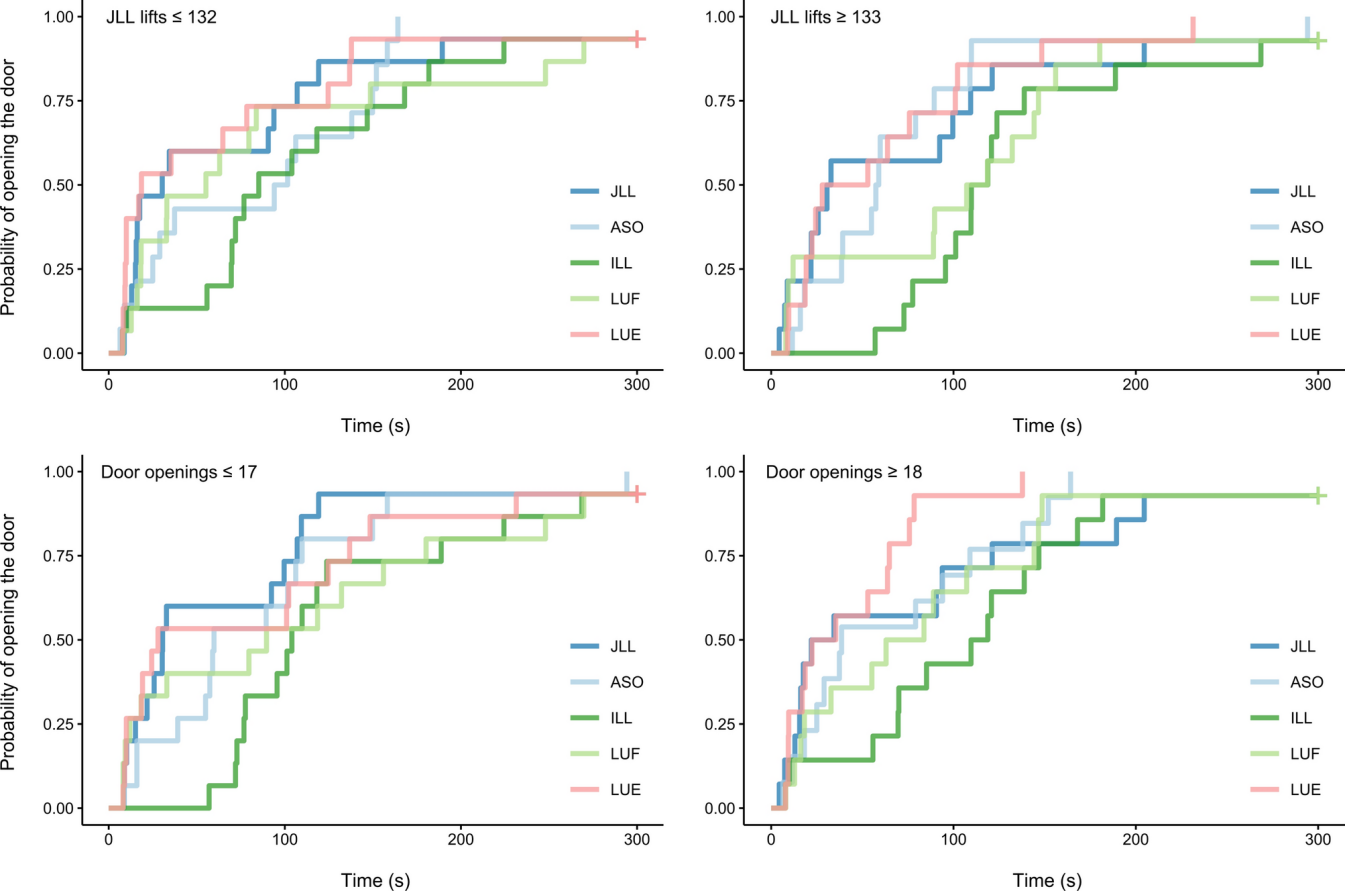
Cumulative incidence plots for the probability of opening the door from the beginning of the session, across time (s). To visualize interaction effects, data is split into two groups in the upper row: subjects that had succeeded with the JLL task fewer than or equal to 132 times (the median), and those that had succeeded with the JLL task greater than or equal to 133 times, in the learning phase. Similarly, in the lower row, data is plotted separately for subjects that had opened the door fewer than or equal to 17 times (the median), and those that had opened the door greater than or equal to 18 times, in the learning phase. *ASO* asocial condition, *JLL* joint log-lift condition, *ILL* individual log-lift condition, *LUE* log up – empty condition, *LUF* log up – full condition.

The full-null model comparison revealed a significant effect of condition or its interactions on the latency to open the door from the beginning of the test session (full-null model comparison: χ^2^ = 17.007, *df* = 4, *P* = 0.002; see Fig. 3). Given that it was unclear whether the interactions or the main effects were responsible for this significant effect, we compared a reduced model lacking the interactions, with the full model (again using a likelihood ratio test). This indicated a trend for the interactions (i.e. a p-value between 0.05 and 0.1; χ^2^ = 14.278, *df* = 8, *P* = 0.075). The model output indicated that this effect was due to both interactions (i.e. between condition and the number of times subject had lifted the JLL in the learning phase and between condition and the number of times the subject had opened the door during the learning phase; see Table 1). An increasing number of lifts of the JLL in the learning phase resulted in a reduction in the latency to open the door in the ASO condition relative to that in the JLL condition. Similarly, with more door openings in the learning phase the latency to open the door in the ILL and LUE conditions declined relative to that in the JLL condition. There was no indication that the latency in the JLL condition changed based on number of lifts or number of door openings in the learning phase.

**Table 1:** Full model output for Cox mixed effects regression model analysing the latency to open the door from the beginning of the session (week 1), including interactions.

Term	Estimate	exp (Estimate)	SE (Estimate)	Z	P
Condition (ASO)^(1)^	− 0.085	0.919	0.290	− 0.29	0.770
Condition (ILL)^(1)^	− 0.934	0.392	0.299	− 3.13	**0.002**
Condition (LUE)^(1)^	0.320	1.378	0.296	1.08	0.280
Condition (LUF)^(1)^	− 0.505	0.604	0.300	− 1.68	**0.093**
Lifts^(2)^	− 0.351	0.704	0.255	− 1.38	0.170
Door openings^(2)^	− 0.339	0.713	0.261	− 1.30	0.190
Condition order^(2)^	− 0.049	0.952	0.088	− 0.56	0.570
Condition (ASO):Lifts	0.594	1.812	0.284	2.10	**0.036**
Condition (ILL):Lifts	0.031	1.032	0.281	0.11	0.910
Condition (LUE):Lifts	0.420	1.522	0.280	1.50	0.130
Condition (LUF):Lifts	0.311	1.365	0.292	1.06	0.290
Condition (ASO):Door openings	0.423	1.527	0.295	1.43	0.150
Condition (ILL):Door openings	0.590	1.804	0.306	1.93	**0.054**
Condition (LUE):Door openings	0.874	2.396	0.294	2.97	**0.003**
Condition (LUF):Door openings	0.398	1.489	0.298	1.34	0.180

*ASO* asocial condition, *JLL* joint log-lift condition, *ILL* individual log-lift condition, *LUE* log up – empty condition, *LUF* log up – full condition, *exp* exponentiated, *SE* standard error. ^(1)^Dummy coded with JLL being the reference category. ^(2)^z-transformed to mean = 0 and sd = 1; mean and standard deviation of the original “lifts” variable were 147.24 and 107.57, for the original “door openings variable” they were 17.13 and 5.69, and for the original “condition order” variable they were 3.01 and 1.42. P-values showing significance or a trend are formatted in bold.

Given that the overall effect for the interactions was only a trend (i.e. a p-value between 0.05 and 0.1), we also report the main effect of condition for a model without the interactions. The main effect of condition was significant according to a comparison of a model lacking the interactions (but containing the main effect of condition) with a model lacking condition and its interactions (χ^2^ = 17.007, *df* = 4, *P* = 0.002). Post-hoc pairwise comparisons indicated that this was due to a longer latency to open the door in the ILL condition than in the ASO, JLL, and LUE conditions (see Table 2).

**Table 2:** Pairwise comparisons of levels of the fixed effect “condition” for reduced Cox mixed effects regression model assessing latency to open the door (i.e. model with interactions omitted; week 1).

Comparison	Estimate	SE	Z	P
ASO – ILL	0.770	0.292	2.637	**0.064**
ASO – JLL	− 0.093	0.292	− 0.317	0.998
ASO – LUE	− 0.370	0.282	− 1.308	0.686
ASO – LUF	0.351	0.291	1.207	0.747
ILL – JLL	− 0.862	0.298	− 2.888	**0.032**
ILL – LUE	− 1.139	0.293	− 3.881	**0.001**
ILL – LUF	− 0.418	0.290	− 1.442	0.600
JLL – LUE	− 0.277	0.297	− 0.933	0.884
JLL – LUF	0.444	0.297	1.494	0.566
LUE – LUF	0.721	0.290	2.490	**0.093**

*ASO* asocial condition, *JLL* joint log-lift condition, *ILL* individual log-lift condition, *LUE* log up – empty condition, *LUF* log up – full condition, *SE* standard error. P-values showing significance or a trend are formatted in bold.

#### Latency to open the door from the last proximity to the box

The full-null model comparison revealed no effect of condition or its interactions on the latency to open the door from the last moment the subjects were in proximity to the box (full-null model comparison: χ^2^ = 14.192, *df* = 12, *P* = 0.289; see Supplementary material ESM_1, Supplementary Fig. 5).

#### Latency to reach proximity to the box after opening the door

The full-null model comparison revealed no effect of condition or its interactions on the latency to reach proximity to the box after opening the door (full-null model comparison: χ^2^ = 13.986, *df* = 12, *P* = 0.302; see Supplementary material ESM_1, Supplementary Fig. 5).

### Week 2

#### Probability of opening the door before reaching the box

The full-null model comparison revealed no effect of condition or its interactions on the probability of opening the door before reaching proximity to the box (full-null model comparison: χ^2^ = 0, *df* = 3, *P* = 1).

#### Latency to open the door

**Fig. 4:**
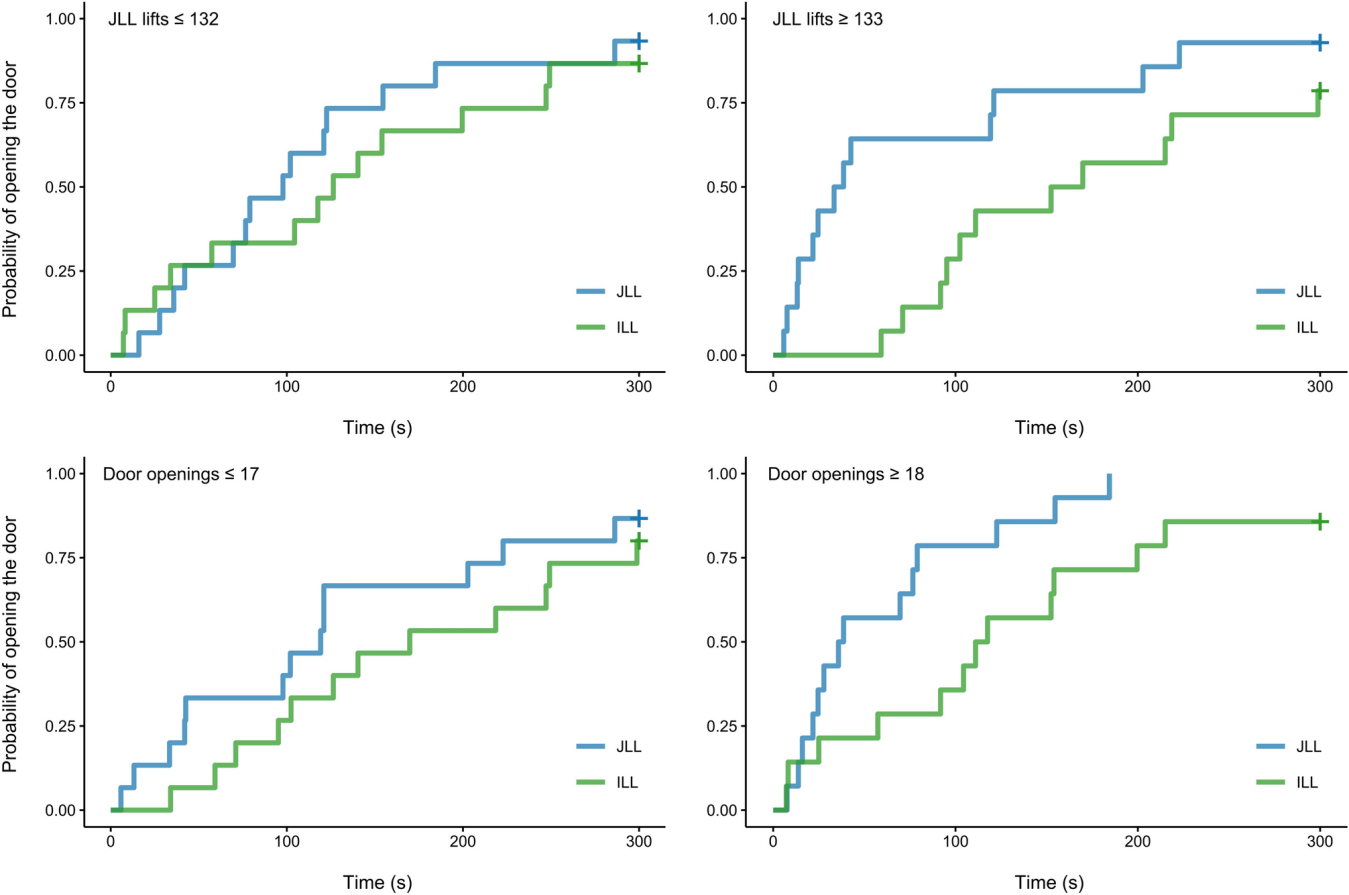
Cumulative incidence plots for the probability of opening the door from the beginning of the session, across time (s). To visualize interaction effects, data is split into two groups: subjects that had succeeded with the JLL task fewer than or equal to 132 (the median), and those that had succeeded with the JLL task greater than or equal to 133 times, in the learning phase. Similarly, in the lower row, data is plotted separately for subjects that had opened the door fewer than or equal to 17 times (the median), and those that had opened the door greater than or equal to 18 times, in the learning phase. *JLL* joint log-lift condition, *ILL* individual log-lift condition.

The full-null model comparison indicated that condition or its interactions had a significant effect on the latency to open the door from the beginning of the test session (full-null model comparison: χ^2^ = 11.789, *df* = 3, *P* = 0.008; see Fig. 4). Comparison of a reduced model, lacking the interactions, with the full model indicated a trend for the interactions (χ^2^ = 5.675, *df* = 2, *P* = 0.059). Assessment of model output revealed that, with an increasing number of successful JLL lifts in the training phase, the latency to open the door in the JLL condition decreased relative to that in the ILL condition (see Table 3). There was also a trend for the latency to open the door in the JLL condition to decrease as the number of lifts of the JLL in the training phase increased (i.e. with more experience of lifting the log successfully in the learning phase, they were quicker to open the door in the test; Estimate = 0.460, SE(Estimate) = 0.251, *P* = 0.067). No such effect was observed for the ILL condition (Estimate = -0.270, SE(Estimate) = 0.246, *P* = 0.270).

**Table 3:** Model output for Cox mixed effects regression model analysing the latency to open the door from the beginning of the session (week 2) including full model with interactions and reduced model presenting main effect of condition.

Term	Estimate	exp (Estimate)	SE (Estimate)	Z	P
Full model output					
Condition (ILL)^(1)^	− 0.889	0.411	0.316	− 2.82	**0.004**
Lifts^(2)^	0.460	1.584	0.251	1.83	**0.067**
Lifts (relevelled: ILL)^(3)^	− 0.270	0.763	0.246	− 1.10	0.270
Door openings^(2)^	0.521	1.683	0.264	1.97	**0.049**
Condition order^(2)^	− 0.061	0.941	0.158	− 0.39	0.700
Condition (ILL):lifts	− 0.731	0.482	0.309	− 2.36	**0.018**
Condition (ILL):door openings	− 0.222	0.801	0.313	− 0.71	0.480
Reduced model output with main effects only					
Condition (ILL)^(1)^	− 0.793	0.453	0.313	− 2.54	**0.011**
Lifts^(2)^	0.038	1.038	0.184	0.20	0.840
Door openings^(2)^	0.344	1.410	0.186	1.85	**0.064**
Condition order^(2)^	0.004	1.004	0.153	0.03	0.980

*ILL* individual log-lift condition; *exp* exponentiated, *SE* standard error. ^(1)^Dummy coded with JLL being the reference category. ^(2)^z-transformed to mean = 0 and sd = 1; mean and standard deviation of the original “lifts” variable were 146.76 and 107.91, for the original “door openings” variable they were 17.17 and 5.73, and for the original “condition order” variable they were 6.5 and 0.50. ^(3)^Relevelled so that ILL became the reference category to assess the effect of number of lifts of the JLL in the learning phase on latency to open the door in the ILL condition. P-values showing significance or a trend are formatted in bold.

Given that the overall effect for the interactions using the likelihood ratio test was only a trend, we also report the main effect of condition for a model without the interactions. The latency to open the door in the JLL condition was shorter than that in the ILL condition (JLL – ILL comparison: Estimate = -0.793, SE(Estimate) = 0.313, *P* = 0.011; see Table 3).

#### Latency to open the door from the last proximity to the box

There was no effect of condition or its interactions on the latency to open the door from the last moment the subjects were in proximity to the box (χ^2^ = 0.757, *df* = 3, *P* = 0.860; see Supplementary material ESM_1, Suplpementary Fig. 6).

#### Latency to reach proximity to the box after opening the door

There was no effect of condition or its interactions on the latency to reach proximity to the box after opening the door (χ^2^ = 1.607, *df* = 3, *P* = 0.658; see Supplementary material ESM_1, Supplementary Fig. 6).

#### Latency to succeed in the JLL condition

**Fig. 5:**
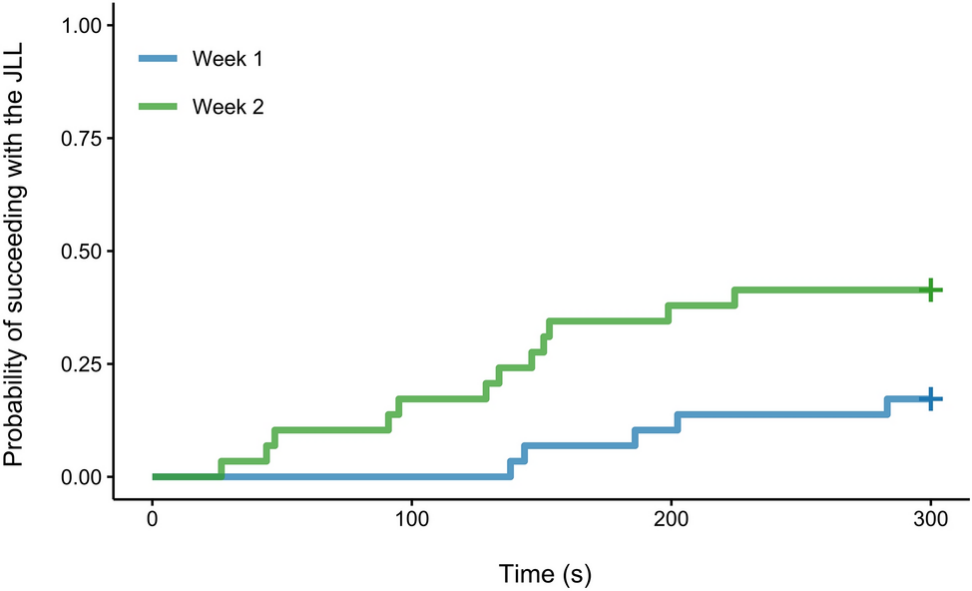
Cumulative incidence plot for the probability of succeeding with the JLL across time (s).

An effect of week on the latency to succeed in the JLL condition was detected (*P* = 0.029; see Fig. 5 and Table 4). The latency to succeed in lifting the log with the partner in the JLL condition was shorter in week 2 than in week 1.

**Table 4:** Full model output for fixed effect for Cox mixed effects regression model assessing effect of week on the latency to succeed in the JLL condition.

Term	Estimate	exp (Estimate)	SE (Estimate)	Z	P
Week 2^1^	1.300	3.671	0.596	2.18	**0.029**

*exp* exponentiated, *SE* standard error. ^1^Dummy coded with week 1 being the reference category. Significant p-values are formatted in bold.

## Discussion

The results of the current study provide limited evidence that pigs understand the need for a partner to help them succeed in the JLL task. All subjects were successful in opening the door in at least one experimental session with 92.61% of subjects on average succeeding per test day. However, their latency to open the door (from the beginning of the session or after proximity to the box) and to return to the box after opening the door, did not differ across conditions in a manner indicative of an understanding of the need for a partner. Following extra exposure to the JLL task and the ILL task in the test pen, this pattern remained largely unchanged, though success with the JLL task increased.

One pertinent significant difference that did emerge in this study occurred in the first test week and was replicated in week 2: subjects took significantly longer to open the door in the ILL condition than in the JLL, ASO, and LUE conditions and the JLL condition in week 2. Given that the latency in the JLL condition did not differ significantly from the other control conditions (in week 1), this significantly longer latency in the ILL is unlikely to indicate an understanding of the need for a partner. It is more plausible that, upon reaching the box, subjects haphazardly attempted to lift the log, which, in the ILL condition, would most likely have resulted in a successful lift and consumption of food treats, potentially causing a delay in leaving the box to open the door. Interestingly, the LUF condition, the only condition with freely available food, did not differ significantly from the ILL condition with regards to the latency to open the door, supporting the idea that food acquisition and consumption did indeed play a role in this delay.

Unlike the latency to open the door from the beginning of the session, the latency to open the door following the last moment the subject was in proximity to the box should have been unaffected by distractions such as log lifting and food consumption. Therefore, it is a more appropriate measure for assessment of subjects’ understanding of the task. Analysis of this latency revealed no evidence for an understanding of the need for a partner. It is also worth noting, if a subject opened the door *before* reaching the box, then no latency to open the door *after* having reached proximity to the box was obtained for this session (as the door would already have been opened). Subjects that understood the need for a partner in the JLL condition could have opened the door before reaching the box, and would thereby fail to produce a latency for the current analysis. However, we did not observe significant differences in the probability of opening the door before reaching the box across conditions.

It must be acknowledged that subjects had differing levels of proficiency with both log lifting and door opening, given that we did not train them to perform these behaviours in a standardized number of training sessions or trials. We accounted for these differing proficiencies by including subjects’ learning phase success at lifting the (JLL) log, and opening the door, as variables in our models, in an interaction with condition. We expected pigs that were more proficient in log lifting and door opening in the learning phase would be more likely to understand the need for a partner and, as a consequence, would be more likely to have shorter latencies to open the door, or to return to the box after opening the door, in the JLL condition relative to other conditions. In week 1, there was evidence that the number of times a subject lifted the log and opened the door in the learning phase influenced the latency to open the door from the beginning of the session, though not in the JLL condition specifically. Thus, there was no evidence that previous experience had an impact on understanding in week 1. In week 2, however, prolificacy in the JLL task during the learning phase appeared to predict the latency to open the door in the JLL condition but not the ILL condition. Subjects with more successful lifts of the JLL during the learning phase tended to be quicker to open the door in the JLL condition specifically. This result is consistent with an understanding that a partner is needed in the JLL condition and not the ILL condition. However, this result should be interpreted with caution, as it is not supported by the other analyses in week 2. Moreover, without the additional control conditions in week 2 that were included in week 1, our ability to draw conclusions regarding effects observed in the JLL condition is restricted. This outcome nonetheless supports the use of more experienced or more highly trained pigs in future investigations of JLL task understanding.

In general, our results corroborate those of Koglmüller et al.^40^ in which Kune Kune pigs failed to wait for a delayed partner to help them with the JLL task, suggesting they did not understand the need for a partner. However, the results of our recruitment approach and Koglmüller et al.’s^40^ delay condition appear to contradict the results of Rault et al.^39^, who observed that pigs’ number of attempts to lift the log in the presence of a partner increased whereas the number of attempts in the absence of a partner decreased over nine days of exposure to the apparatus. A decline in number of attempts overall was not observed. These results suggest that the pigs learned when to act appropriately, though it may be that the pigs learned to respond to specific local cues without recognizing the presence of the partner as an essential prerequisite. It should be acknowledged though that our study set a relatively high bar, requiring that subjects not only understand the need for a partner but also that they understand various aspects of the setup.

There were at least two important assumptions in the current study that deserve a mention. First, the design assumed that the pigs’ primary motivation in the test setting was to obtain the food. However, there could have been multiple competing motivations. In particular, when faced with inaccessible food treats, rather than attempting to achieve access, subjects’ motivation to obtain food from the box may have quickly lapsed, with other competing motivations taking over. In this context, it is worth considering that even if the pigs were capable of understanding that they need a partner and understood how to recruit a partner, their self-control capacities could have precluded them following the required steps to achieve this outcome. A number of studies have investigated delayed gratification in pigs, with a maximum wait of 50 s for a larger quantity of food for pigs that were of similar age to ours, though considerable variation was observed among individuals and pigs that were older than the age range of ours (14 – 16 weeks of age) were shown to be capable of withstanding longer delays than pigs in the age range of 7 – 9 weeks^65–67^. The context of our study may differ from these delay of gratification studies too much, however, to allow strong conclusions regarding the role played by self-control.

A second assumption is that the pigs understood the consequences of opening the door. We did not test for this understanding explicitly. In Range et al.’s^37^ and Melis et al.’s^47^ studies on recruitment, the subjects’ understanding of the consequence of opening the door was implied by the fact that the subjects performed this behaviour in a context-appropriate manner. Had our subjects also behaved in this manner (i.e. recruiting a partner only when they were needed), further verification of their understanding of the significance of opening the door would not be essential. Interestingly, Range et al.^37^ and Melis et al.^47^ both initially provided their study participants the opportunity to learn the door-opening procedure in the absence of a partner (i.e. opening the door did not result in recruitment of a partner); however, mid-experiment, in an ad hoc learning phase, they both introduced a new procedure in which opening the door did result in recruitment of a partner. In both cases, subjects’ performance in the test setting improved after this additional learning phase. Although our door-opening learning phase was carried out in a social setting, perhaps providing more opportunities for the pigs to learn specifically that opening the door allows a partner to enter their enclosure, would have facilitated success in the test situation. It is noteworthy that the probability of interacting with the handle of the real door was significantly higher than the probability of interacting with the two decoy handles (see Supplementary material ESM_1). This could indicate that the subjects knew which door handle was the correct one and that they interacted with the door handle with the intention of opening the door. However, given their greater experience with this handle and its more frequent use by the pigs in general, we cannot rule out alternative explanations for the greater probability of interacting with the real handle such as familiarity or local enhancement.

With regards to learning about the JLL task, realizing the necessity of a partner is conceivably more difficult than in other analogous tasks. In the loose-string task^38,47^, for example, both ends of the string/rope typically extend into an enclosure such that cooperating partners can see both ends and can see each other when pulling. In the JLL task, the log sits inside the box into which pigs must insert their snout. It is likely that, when lifting the log, a pig cannot see what the partner is doing. Future variants of the JLL task could take this feature into consideration and, in doing so, could also increase the comparability of the JLL task with other cooperation tasks, though this issue is tied to the anatomy of the pig and the characteristics of the task.

Particularly noteworthy also is the fact that the pigs participating here were very young. Studies investigating the upper limits of non-human animal cognition tend to focus on adults rather than juveniles, though a sizable proportion of cognitive studies on pigs employ juveniles (see Gieling et al.^68^ for review). Studies on pigs’ cognitive development are scarce, though Krause et al.^67^ found shorter waiting times in 9-week-old than 16-week-old pigs in a delay of gratification task, demonstrating not only that age influences performance in a cognitive task but also that even a 7-week age difference can result in a significant change. Nevertheless, pigs show remarkable cognitive abilities already at a very young age, such as the ability to establish a teat order at suckling within the first 4 days after birth^69^. Insights from the broader comparative cognition literature further support the idea that age can influence the response in cognitive tasks. For example, 8- to 10-week old assistance dog puppies were recently found to perform more poorly than do adults on a cognitive test battery^70^. Moreover, the performance of these subjects was shown to have increased at 21 months of age^71^. To draw conclusions about pigs’ ability to understand the need for a partner in cooperative tasks, it would be worthwhile to incorporate the study of older animals.

It is also worth highlighting that the choice of dyads for testing could have had an impact on the outcome of our study. Rather than choosing dyads that had very high success rates together, we paired dyads that had performed together a specific minimum number of times, to allow us to maximize the sample size without changing the partner. Recent literature underpins the notion that the social relationship between partners may be crucial in predicting cooperative outcomes. In a review of performance of wolves in cooperative tasks, Dale et al.^72^ found social relationship between individuals in a dyad to have a greater impact on the outcome than cognitive factors. Moreover, Rault et al.^39^ observed that successful pigs appeared to form preferential dyadic associations to solve the JLL task, suggesting that specific individuals are more likely to succeed together. It is possible that during the learning phase of our study, very high success rates occurred between individuals with a strong social relationship. Strong relationships may have developed, in turn, due to frequent co-lifting. The absence of the preferred partner during our tests conceivably diminished subjects’ desire to engage with the task. In a similar vein, subjects may have associated success with a specific partner; thus, in the absence of their favoured partner, the JLL task may have seemed unsolvable. Pairing individuals together that had very high success rates across the learning phase could, therefore, have resulted in a different outcome. It is important to note, nonetheless, although the number of successful JLL lifts during the learning phase ranged from one to 86 within the selected test dyads, for these participants the average number of successful JLL lifts (with any partner) during the learning phase was 147. Therefore, competence at the task was high in general.

Finally, it is important to emphasize, overall, that our results do not prove that the pigs did not understand the need for a partner, but rather fail to provide clear evidence that they did understand. At least some pigs in the sample may have opened the door to recruit the partner appropriately. However, our analyses were focused on the group level and the absence of repeated testing precluded evaluation of individual consistency in performance, which would have been required to infer such an understanding at the individual level. Moreover, the design of the study relied on latencies differing across conditions based on the pigs’ decision in each condition. It is possible that the same latency could emerge in different conditions for different reasons. However, a more parsimonious explanation is that the pigs simply did not differentiate between the conditions.

In conclusion, although the pigs in this study were very successful at lifting a log with a partner over the learning phase, there was only limited evidence from the subsequent test situation that they understood the need for a partner. Despite opening the door in the test enclosure and in some cases succeeding in lifting the log with the partner in the JLL condition, the comparison of conditions for the most part failed to indicate that they adjusted their behavior to whether a partner was needed or not. Future work will be required to determine whether pigs understand the consequences of opening a door in this kind of setup. Testing older individuals will also help to shed light on pigs’ level of understanding of cooperative interactions. Finally, in future studies, pairing individuals that have succeeded frequently together during the learning phase could produce a different outcome.

## Supplementary Information

Supplementary Information 1.

Supplementary Video 1.

Supplementary Information 2.

## Data Availability

The dataset analyzed in the current study is included in the electronic supplementary materials.
