# Supplementary material for: Pigs solve a cooperative task without showing a clear understanding of the need for a partner
Source: Sci Rep. 2025 Feb 11;15:5095. doi: 10.1038/s41598-024-84529-3 (PMC11814144; doi:10.1038/s41598-024-84529-3)
Supplement: Supplementary file 1 — Supplementary Information 1. [file 41598_2024_84529_MOESM1_ESM.pdf]

Supplementary Material:

Pigs solve a cooperative task without showing a clear understanding of the need for a partner

Jim McGetrick[1,2]*‡, Kimberly Brosche[1]‡, Clémence Nanchen[1], Jean-Loup Rault[1]

[1]Institute of Animal Welfare Science, University of Veterinary Medicine, Vienna, Veterinärplatz 1, 1210 Vienna, Austria

[2]Department of Behavioral & Cognitive Biology, University of Vienna, Djerassiplatz 1, 1030 Vienna, Austria

*Corresponding author: james.mcgetrick@univie.ac.at

‡These authors contributed equally

Table of Contents

1. **Diagrams of joint log-lift (JLL) box** 2
2. **Analysis of learning the JLL and individual log-lift (ILL) tasks** 3
3. **Photographs of test enclosure** 6
4. **Analysis of door openings during the learning phase** 7
5. **Details of test dyads** 9
6. **Cumulative incidence plots** 10
7. **Analysis of the probability of interacting with the door handles** 13
8. **Pilot study** 15
9. **References** 20

1. Diagrams of joint log-lift (JLL) box

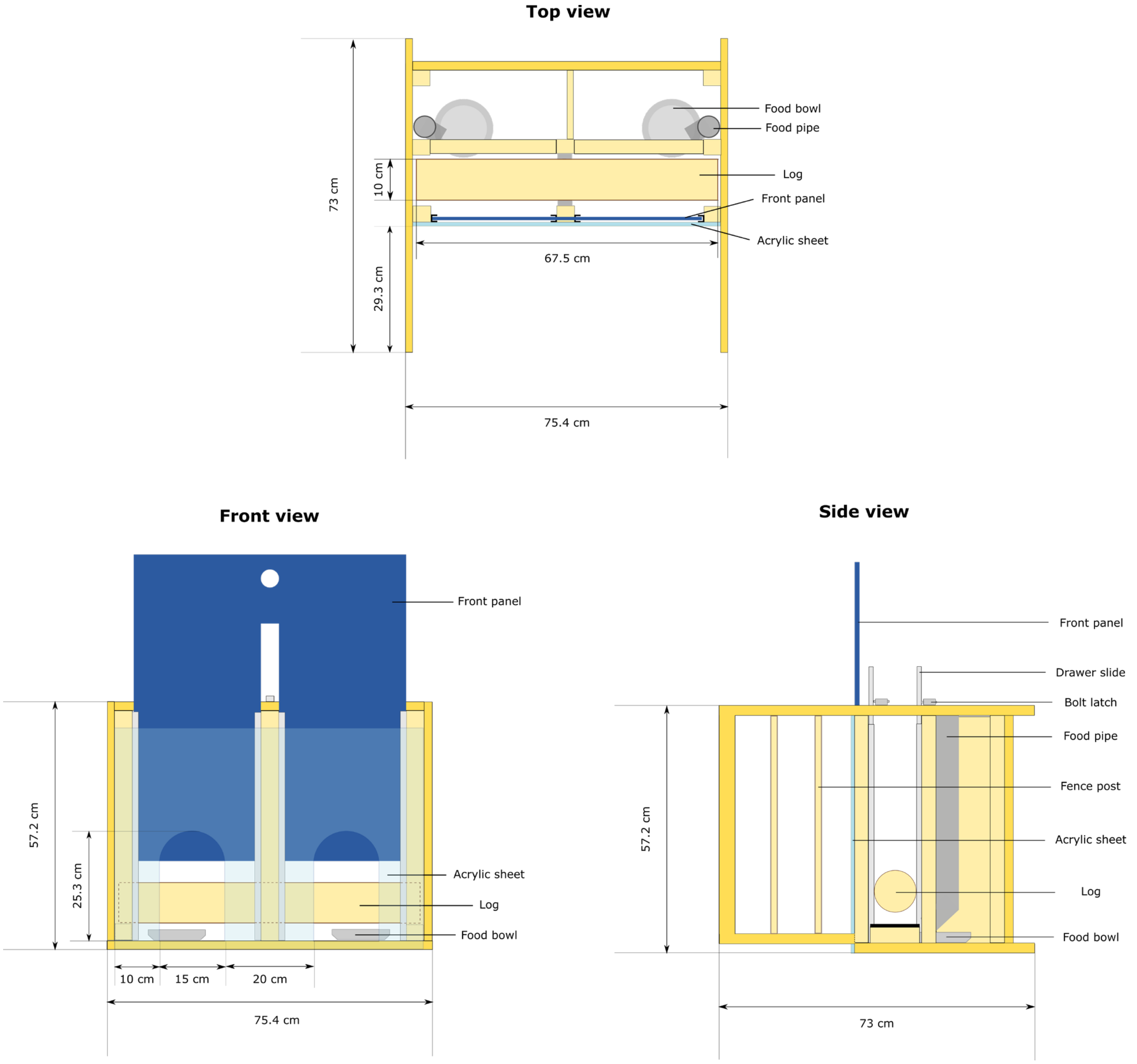

Supplementary Figure 1 Diagrams of the JLL box, viewed from the top, front and side.

2. Analysis of learning the JLL and individual log-lift (ILL) tasks

2.1. Methods

To assess changes in log-lifting performance across the first 10 days of the log-lift learning phase, we fitted a Generalized Linear Mixed Effects Model (GLMM[1]) with a Poisson error distribution. Number of successful lifts was included as the response variable. We included fixed effects of box type (i.e. JLL, ILL), day, and day-squared with an interaction between box type and day and between box type and day-squared.

We included a random intercept effect of group. Within the random effect of group, the random slopes of box type, day and day-squared were included, with the same interactions mentioned for the fixed effects[2,3]. Prior to inclusion as a random slope, box type was manually dummy coded and centred.

We fitted the model using the function “glmer” from the package “lme4” (version 1.1-23[3]). Prior to fitting the model, we z-transformed day to a mean of zero and a standard deviation of one to allow for an easier interpretation of results and to ease model convergence.

This model was overdispersed (dispersion parameter: 1.682). To reduce this dispersion parameter slightly we included a random intercept effect representing each individual observation (hereafter “observation level random effect”). This reduced the dispersion parameter though the model was still overdispersed (final dispersion parameter: 1.367). No random slopes were included within the observation level random effect.

To rule out collinearity, we determined Variance Inflation Factors (VIFs[4]) using the function “vif” from the package “car” (version 3.0-8[5]). This was applied to a model lacking day-squared, interactions and random effects. Collinearity did not appear to be an issue (maximum VIF: 1). We confirmed that the best linear unbiased predictors (BLUPs[1]) did not deviate from a normal distribution, by visual inspection of histograms[1,6].

To obtain confidence intervals, we used a parametric bootstrap (function bootMer of the package “lme4”; N = 1,000 bootstraps).

As an overall test of the effect of condition, we conducted a full-null model comparison[7] whereby all fixed effects were excluded from the model. The sample for the analysis included a total of 120 observations across 6 groups.

2.2. Results

The full model differed significantly from the null model (full-null model comparison: χ^2 = 29.1836, *df* = 5, *P* < 0.001). Applying the “drop1” function in R revealed a significant interaction between box type and day and a trend for the interaction between box type and day-squared. The number of successful lifts across the 10-day period increased for both boxes though there was a slightly sharper increase in performance with the ILL. The number of successful lifts was smaller with the JLL box in general (see **Supplementary Table 1** and **Supplementary Fig. 2**).

Supplementary Table 1 Full model output including estimate, standard error, z-value, p-value and lower and upper 95% confidence intervals for model assessing successful lifts across the learning period. P-values showing significance or a trend are formatted in bold.

Term	Estimate	SE	Z	P[3]	Lower CI	Upper CI
Intercept	4.191	0.049	84.805		4.107	4.286
Day[1]	0.330	0.023	14.302		0.287	0.378
Box (JLL)[2]	-0.731	0.178	-4.104		-0.969	-0.449
Day-squared	-0.122	0.032	-3.882		-0.182	-0.071
Day:Box (JLL)	0.208	0.063	3.295	**0.011**	0.093	0.332
Box (JLL):Day-squared	-0.104	0.050	-2.065	**0.054**	-0.190	-0.018

[1]z-transformed to mean = 0 and sd = 1; mean and standard deviation of the original variable were 5.5 and 2.88
[2]Dummy coded with ILL being the reference category
[3]P-values presented are from the output of the “drop1” function

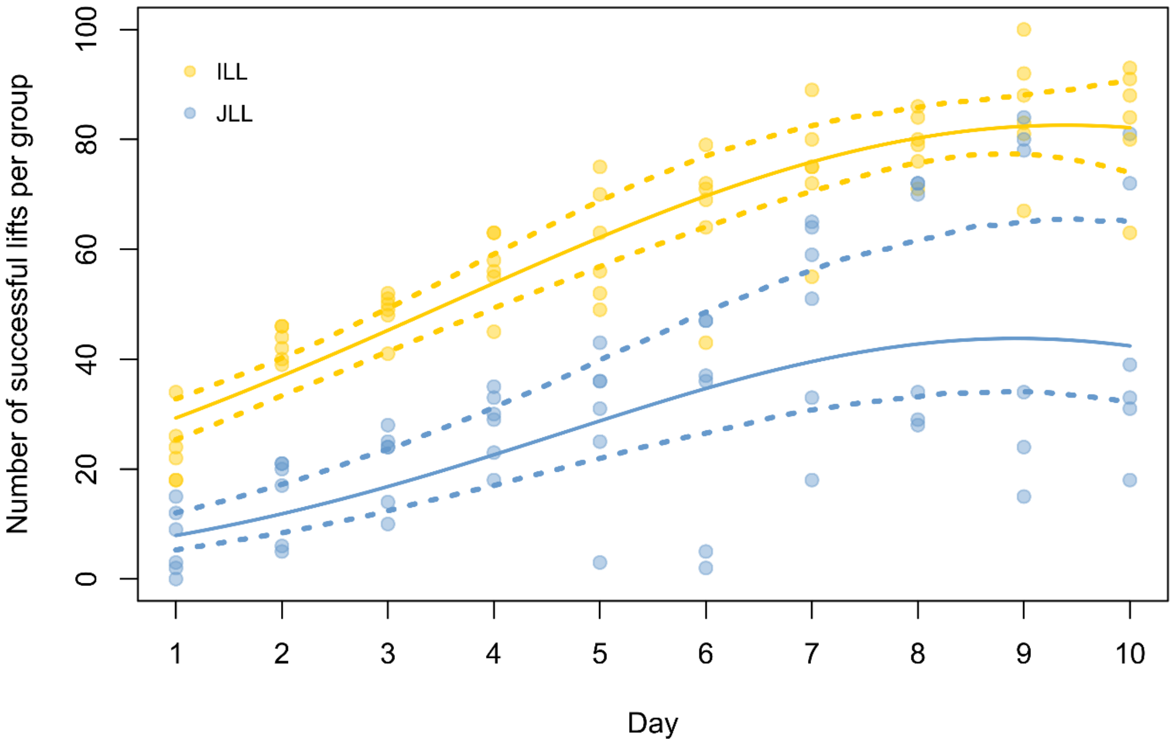

Supplementary Figure 2 Number of successful lifts with the joint log-lift (JLL) box or the individual log lift (ILL) box per group across the first 10 days of the learning phase. Solid lines represent the fitted model; dashed lines represent its 95% confidence intervals.

3. Photographs of test enclosure

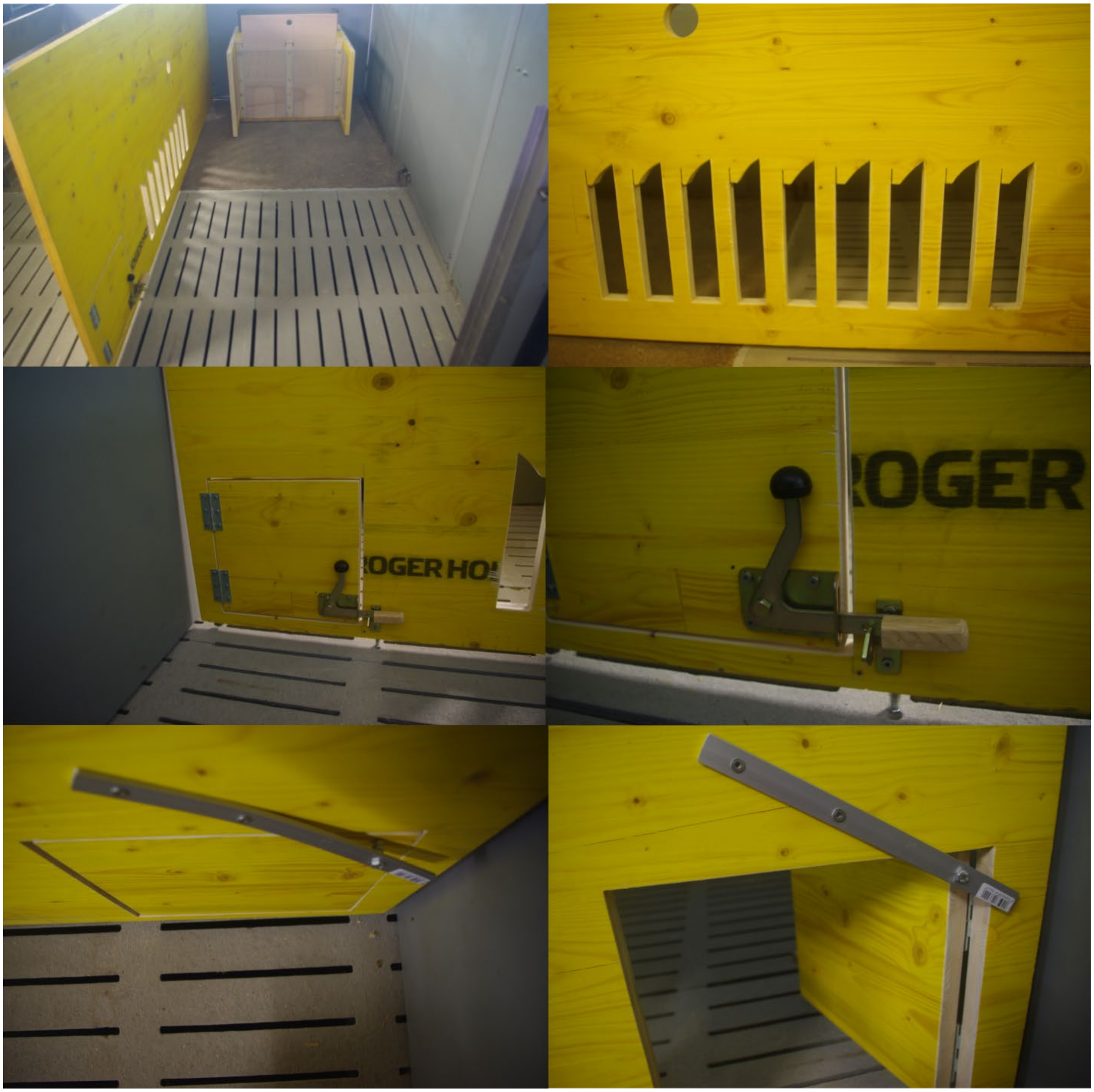

Supplementary Figure 3 Test enclosure with separating wall and its various features. Top left, subject’s enclosure with JLL box present and the separating wall to the left (note: the door of the test enclosure is fully open to allow for a photograph from this perspective); top right, slats in separating wall to allow pigs to see the adjacent compartment; middle left, view of small door from the subject’s compartment; middle right, close up view of the door latch; bottom right, mechanism that causes door to swing open from the partner’s compartment; bottom right, view of open door from partner’s compartment.

4. Analysis of door openings during the learning phase

4.1. Methods

To determine whether pigs' door opening proficiency improved over the course of the 5-day learning period, we fitted a GLMM with a Poisson error distribution. The number of times each pig opened the door was included as the response variable. Day was included as a fixed effect. Group (i.e. the identity of the home group of the individual pig) was included as a random effect. The random slope of day was included within the random effect of group. Prior to fitting the model, we z-transformed day to a mean of zero and a standard deviation of one. The model was fitted using the function "glmer" from the package "lme4" (version 1.1-33[8]). The model was slightly overdispersed (dispersion parameter: 1.316). To obtain confidence intervals, we used a parametric bootstrap (function bootMer of the package "lme4"; N = 1,000 bootstraps).

As an overall test of the effect of day, we conducted a full-null model comparison[7] whereby day was removed from the model to generate a null model. The comparison was based on a likelihood ratio test[9] using the function "anova" and setting the "test" argument to "Chisq". The sample for the analysis included a total of 165 observations across 6 groups.

4.2. Results

The full model differed significantly from the null model (full-null model comparison: χ^2 = 7.6796, df = 1, P = 0.006) with the number of door openings significantly increasing with day (see **Supplementary Table 2** and **Supplementary Figure 4**).

Supplementary Table 2 Full model output including estimate, standard error, z-value, p-value and lower and upper 95% confidence intervals for model assessing number of door openings across the learning period. Significant p-values are formatted in bold.

Term	Estimate	SE	Z	P[3]	Lower CI	Upper CI
Intercept	1.229	0.043	28.90		1.136	1.307
Day[1]	0.205	0.042	4.84	**0.006**	0.120	0.290

[1]z-transformed to mean = 0 and sd = 1; mean and standard deviation of the original variable were 3.00 and 1.42.

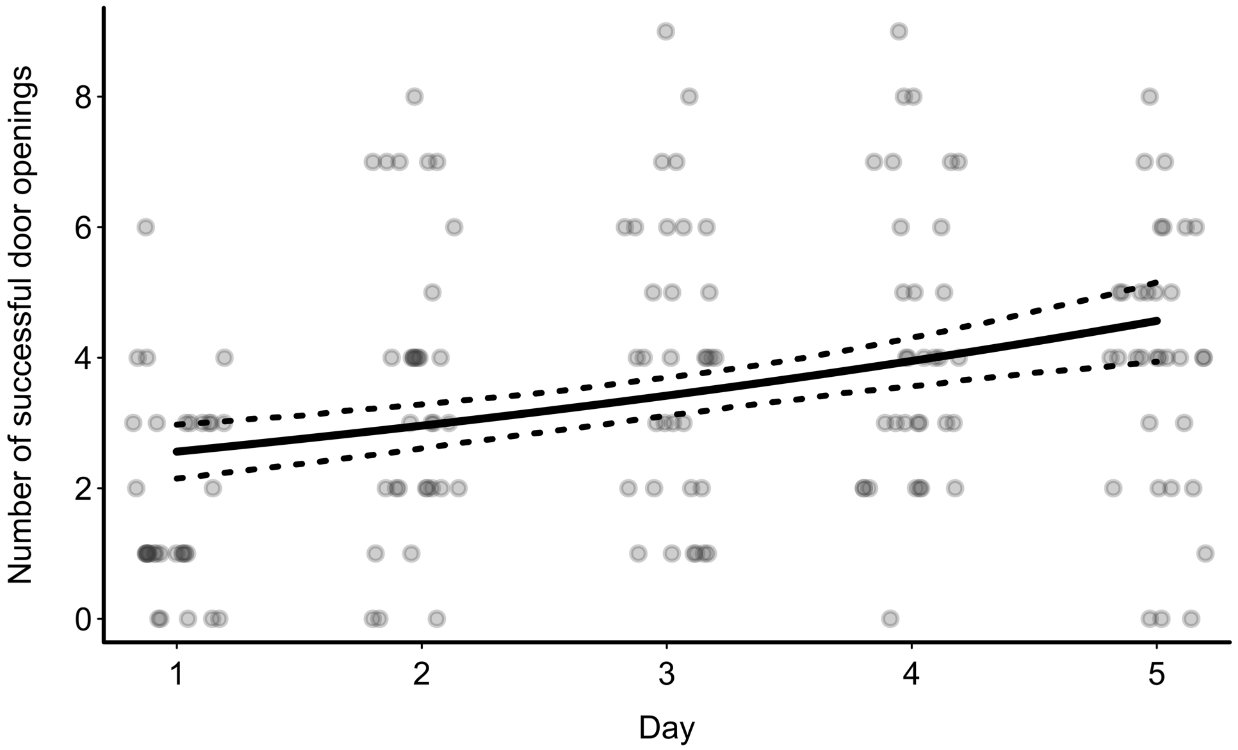

Supplementary Figure 4 Number of door openings across the door-opening learning phase in the test enclosure. Black line represents the fitted model; dashed lines represent its 95% confidence interval.

5. Details of test dyads

Supplementary Table 3 List of test dyads including the number of times they succeeded together with the JLL in the learning phase, as well as their total number of successful lifts of the JLL and total number of successful door openings in the learning phases. Note: successes with the JLL are based on values after day eleven of log-lift learning phase.

Subject	Partner	No. of successes together	Total no. of lifts with JLL (with any other pig)		Total no. of door openings	
			Subject	Partner	Subject	Partner
1bh	1rl	12	78	46	23	16
1gh	1bl	16	30	99	14	27
2gh	2bh	17	80	92	22	19
2gl	2rl	19	48	132	6	21
2rh	2bl	1	17	73	16	15
3bh	3gh	23	49	289	25	14
3gl	3rl	11	30	345	28	20
3rh	3bl	26	133	196	15	10
4gh	4bh*	10	265	26	9	NA
4bl	4rl	74	178	241	12	20
5bh	5gl	26	144	141	14	18
5gh	5rh	27	127	139	9	25
5rl	5bl	12	28	279	18	17
6bh	6rh	28	76	342	19	11
6rl	6bl	86	424	135	24	11

*Not tested as a subject due to not having been given the chance to learn how to open the door in the learning phase

6. Cumulative incidence plots

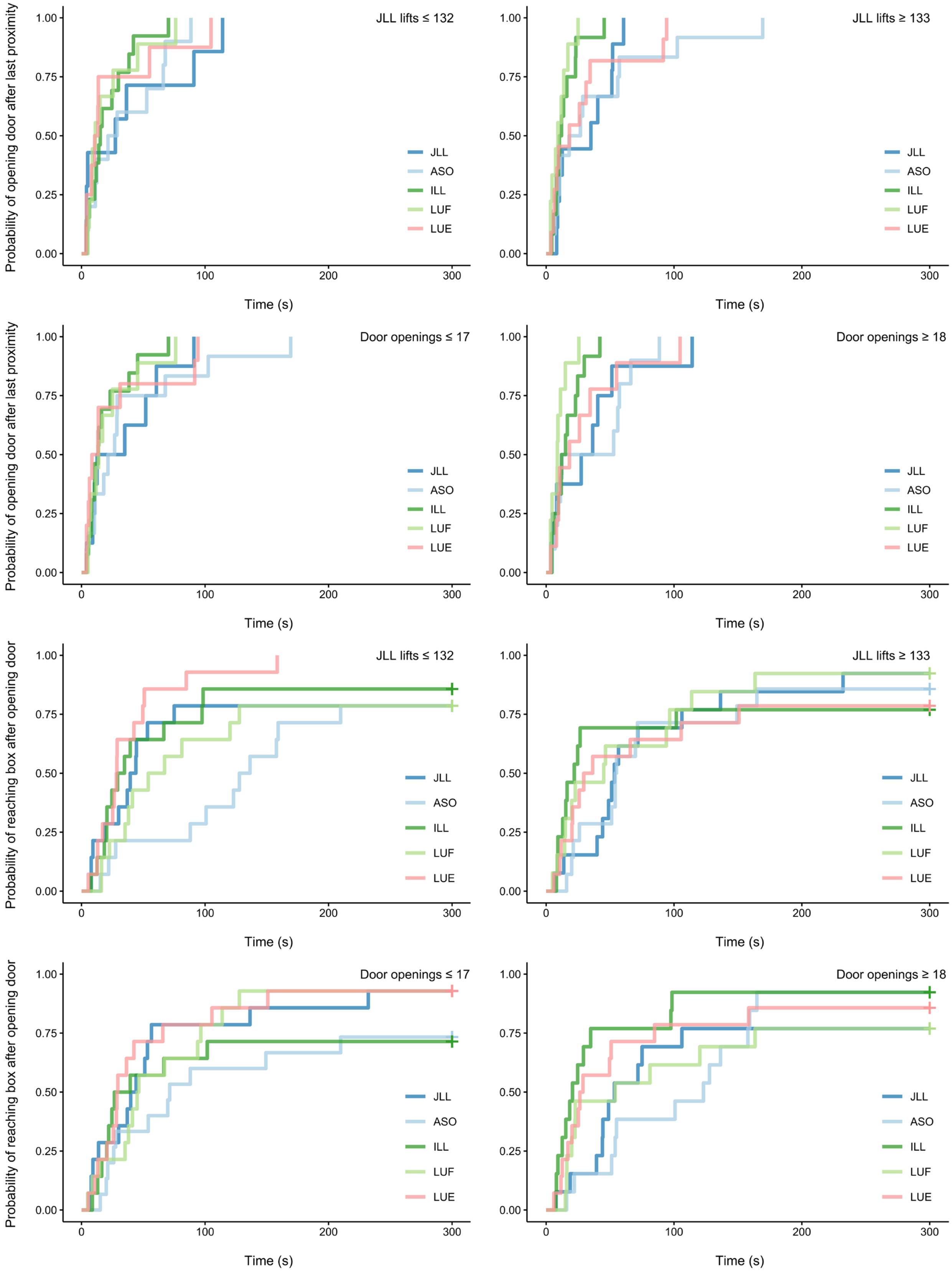

Supplementary Figure 5 Cumulative incidence plots for the probability of opening the door after the last moment the subjects were in proximity to the box (before the door was opened) (top 4 plots), and the probability of reaching proximity to the box after opening the door (bottom 4 plots), across time (s), in week 1. To visualize interaction effects, data is split into two groups in the upper row, for each variable: subjects that had succeeded with the JLL task fewer than or equal to 132 times, and those that had succeeded with the JLL task greater than or equal to 133 times, in the learning phase. Similarly, in the lower row, for each variable, data is plotted separately for subjects that had opened the door fewer than or equal to 17 times, and those that had opened the door greater than or equal to 18 times, in the learning phase. ASO, asocial condition; JLL, joint log-lift condition; ILL, individual log-lift condition; LUE, log up – empty condition; LUF, log up – full condition.

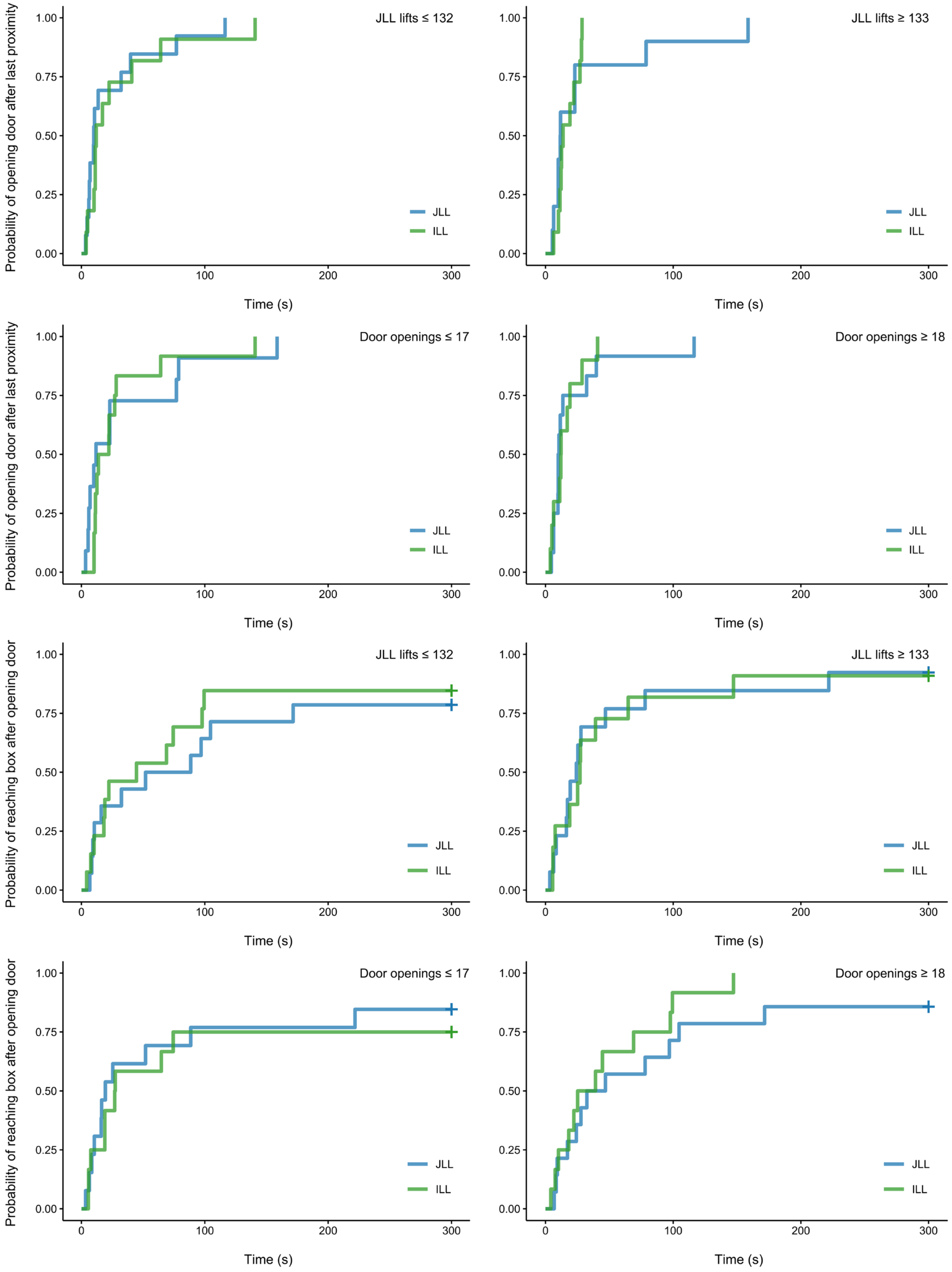
Probability of opening door after last proximity
JLL lifts ≤ 132
JLL lifts ≥ 133
Door openings ≤ 17
Door openings ≥ 18
Probability of reaching box after opening door
JLL
ILL
Time (s)
0.00
0.25
0.50
0.75
1.00
0
100
200
300

Supplementary Figure 6 Cumulative incidence plots for the probability of opening the door after the last moment the subjects were in proximity to the box (before the door was opened) (top 4 plots), and the probability of reaching proximity to the box after opening the door (bottom 4 plots), across time (s), in week 2. To visualize interaction effects, data is split into two groups in the upper row, for each variable: subjects that had succeeded with the JLL task fewer than or equal to 132 times, and those that had succeeded with the JLL task greater than or equal to 133 times, in the learning phase. Similarly, in the lower row, for each variable, data is plotted separately for subjects that had opened the door fewer than or equal to 17 times, and those that had opened the door greater than or equal to 18 times, in the learning phase. JLL, joint log-lift condition; ILL, individual log-lift condition.

7. Analysis of the probability of interacting with the door handles

7.1. Methods

To determine whether pigs were more likely to interact with the real handle than the two decoy handles, in the first test week, we fitted a GLMM with a binomial error distribution and a logit link function[10] (0, did not interact with the handle; 1, interacted with the handle).

We included handle identity (real handle, left handle, right handle) as the main predictor of interest. We included random intercept effects of subject, dyad (i.e. the subject-partner pairing), and group (i.e. the group that the subject was housed in throughout the study period). We fitted the model using the function “glmer” from the package “lme4” (version 1.1-33[8]). Initially, we fitted this model with “condition” as a predictor, in an interaction with handle type. However, most likely due to complete separation, the model estimates were unreliable; therefore, condition was removed from the model and the interaction effect was assessed visually with the plotted data (see below).

As an overall test of the effect of handle, we conducted a full-null model comparison[7]. The null model was an intercept only model. The comparison was based on a likelihood ratio test[9] using the function “anova” and setting the “test” argument to “Chisq”. The sample for this model included a total of 432 observations across 29 subjects, 15 dyads, and 6 groups. Post-hoc pairwise comparisons of the conditions were carried out using the function “emmeans” in the package “emmeans” (version 1.8.6[11]), setting the “specs” argument to “pairwise ~ handle”. Confidence intervals were also obtained using the “emmeans” package.

7.2. Results

The full-null model comparison revealed a significant effect of handle identity on the probability of manipulation by the pigs (full-null model comparison: $\chi^2 = 212.92$, $df = 2$, $P < 0.001$). Posthoc

pairwise comparisons indicate that the probabiilty of interacting with the real handle was significantly higher than the probability of interacting with the two decoy handles (see **Supplementary Table 4** and **Supplementary Fig. 7**). The probability of interacting with the handle to the left (i.e. on the wall separating the subject and partner's enclosure) was also significantly higher than the probability of interacting with the handle on the opposite wall. Based on visual inspection of the plotted data with condition taken into account, no strong condition specific effects were evident (see **Supplementary Fig. 8**).

Supplementary Table 4 Pairwise comparisons of handle identity. SE, standard error. Significant p-values are formatted in bold.

Comparison	Estimate	SE	Z	P
Left Handle – Real Handle	-4.45	0.757	-5.869	**<0.001**
Left Handle – Right Handle	1.31	0.272	4.810	**<0.001**
Real Handle – Right Handle	5.75	0.774	7.432	**<0.001**

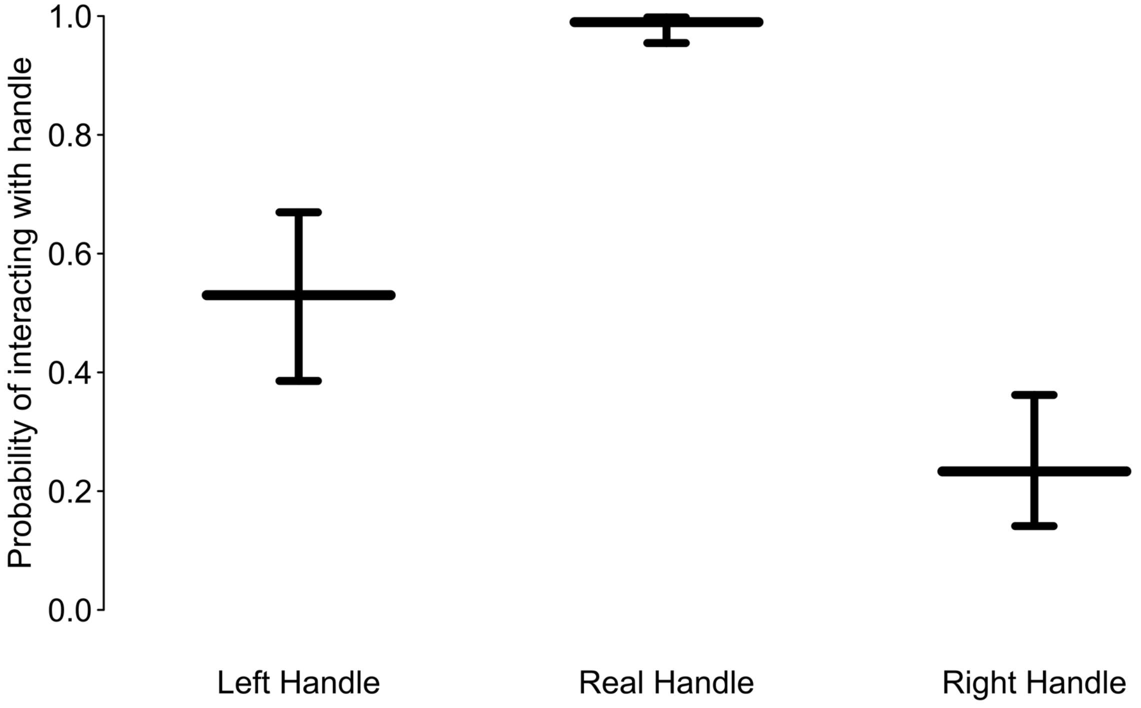

Supplementary Figure 7 Proability of interacting with each handle type in test sessions in the test pen (week 1). Horiztontal bars represent the meodel estimates and whiskers represent the 95% confidence intervals.

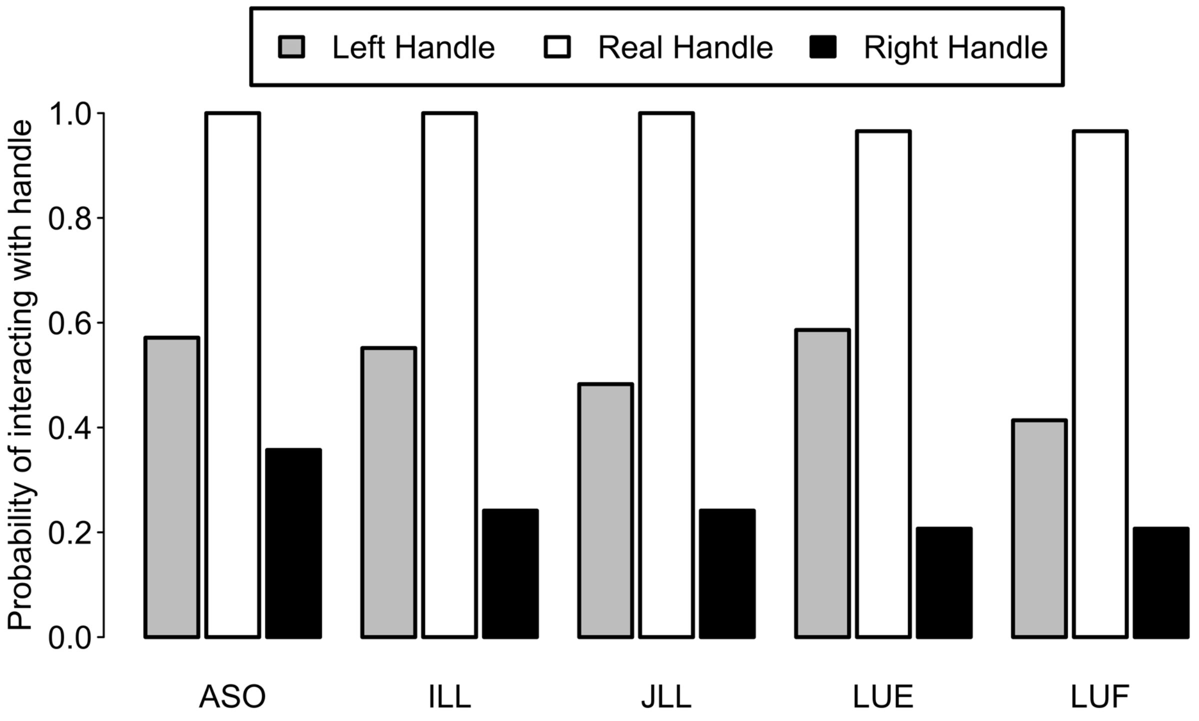

Supplementary Figure 8 Probability of interacting with each handle type in each condition in the test pen (week 1).

8. Pilot study

8.1. Aim

The aim of this study was to pilot a new JLL box and depending on the success, to pilot the test methods to be used in the main study on recruitment of a partner. There were numerous differences between the new box used in this study and the older version used by Rault et al.[12]. The most salient differences included the use of a round log to prevent the pigs' noses from coming into contact with hard edges when lifting, fences on either side of the box so that the pigs interacting with the log could not be physically displaced from the side by other group members, pipes through which the treats could be dropped to bait the food bowls, and a plywood panel at the front of the box which could be lowered or raised to block or provide access to the log, respectively.

8.2. Methods

The methods performed in the pilot study closely matched those for the main study, though the ILL box was not used. Deviations from the procedure used in the main study are outlined here.

8.2.1. Subjects and housing

Sixty pigs (30 females and 30 castrated males) were included in the study. These pigs were selected initially from 11 different litters and were approximately 4 weeks of age at the beginning of the study. The pigs were divided into six groups, each composed of five females and five males. One littermate, of the opposite sex, of each individual in the group was present.

8.2.2. Apparatus

8.2.2.1. Joint log-lift box

The log rested horizontally inside the box and was attached to an immobile central rail which allowed it to move up or down inside the box. Upon lifting the log successfully, it would attach to a magnet inside the box at the upper end of the rails; thus, when the log was lifted to a height of approximately 19 cm (from the floor of the box to the bottom of the log), the log was locked in place. When locked in this position, the log could not be lifted any higher. Upon reaching its highest position (i.e. when it attached to the magnet) a small light on the top of the box was activated so that the experimenter knew the log was locked in place. The experimenter could release the log by pressing a button which resulted in the log being pushed off the magnet.

8.2.3. Log-lift box exposure and learning phase

8.2.3.1. Experimenters

One female experimenter conducted the log-lift box learning phase.

8.2.3.2. Provision of food treats

For the first two days of the study, each group of pigs was provided with pieces of apple, in order to familiarize them with this food type used throughout the study. The experimenter entered the enclosure, with a bowl/handful of apple pieces (each ~1 cm^3) and handed pieces to each pig individually for approximately 15 minutes per group.

8.2.3.3. Exposure to the box

On the subsequent three days, the pigs were exposed to the JLL box in their home enclosure. The front panel of the box was always the same colour.

8.2.3.4. Learning to lift the log

The log-lift learning phase took place over 20 days as per the methods of the main manuscript (in the first week, the box was presented from Monday to Friday, in the second week from Monday to Thursday, in the third week from Sunday to Friday, and in the fourth week from Monday to Friday). From day six of this learning phase, the log was fixed in the upper position for the first 5 minutes of the session so that pigs could access the food treats within. From day 10 onwards, an apple piece was placed under the log on either side to encourage the pigs to attempt to lift the log. These were replaced with new apple pieces by the experimenter each time the pigs lifted the log high enough to retrieve them.

Success in lifting the log together was very low with only six successful lifts in total spread across the first 15 days and with all of these successful lifts being performed by one group (see **Supplementary Fig. 9**). One possible explanation for the low success rate is that the height to which the pigs were required to lift the log was too demanding. Therefore, we decided to lower the height to which the log had to be lifted, by approximately 5.5 cm for days 16 – 20.

It also seemed as though pigs attempting to lift the log within each group may have been too frequently disrupted by other group members pushing in to access the box. Therefore, for days 16 – 20, in addition to continuing sessions in the home enclosure, we selected pigs from each group that had shown promising attempts to lift the log and brought these to the test enclosure (with the separating wall removed) to provide them with the opportunity to lift the log in smaller groups. These small groups comprised two to six pigs of the same home group, that had succeeded in lifting the log with a partner at least once during the previous training phase (4 pigs in group 1, 2 pigs in group 2, 6 pigs in group 3, 4 pigs in group 4, 3 pigs in group 5, 4 pigs in group 6). Each session lasted 15 minutes during which pigs were free to interact with the log and jointly lift it as often as they pleased. The log was reset after each successful attempt. To

additionally enhance pigs' motivation to solve the task, an apple piece was placed under the log on either side by the experimenter, as carried out in the learning phase in the group.

8.3. Results & Discussion

Success in lifting the log together was very low over the first 15 days in the test enclosure and appeared to increase in the last five days (see **Supplementary Fig. 9**). All lifts in the first 15 days came from one group; however, in the last five days, all groups lifted the log at least once in their home enclosure. The greater spread in number of successful lifts across groups may have been due to a reduction in the height to which the log had to be lifted or due to the extra exposure to the box in smaller groups in the test enclosure. Sessions in the test enclosure were relatively successful with all groups lifting the log together at least once and with a maximum number of lifts of 28 by one group in one session (see **Supplementary Fig. 9**).

8.4. Additional changes & conclusions

Overall, based on the experience with this pilot study, we deemed it feasible to conduct a new study to test pigs' understanding of the need for a partner in the JLL task, provided the above changes were implemented. Given the increased success rate after lowering the height to which the log had to be lifted and reducing the number of pigs present during the learning phase, we decided to keep this new adjusted height and to reduce the number of pigs in the home enclosures (from ten to six) for the study presented in the main manuscript. We also decided to change the mechanism by which the log locks in the upper position, from a magnet-based mechanism to the drawer slide-based mechanism described in the main manuscript. The reason for this was twofold. First, the magnet was not reliable: often the log dropped after seemingly having locked in place. Second, with the new lowered height of the log when in its upper position, it was important for the pigs to be able to lift the log higher for ease of access to the food treats.

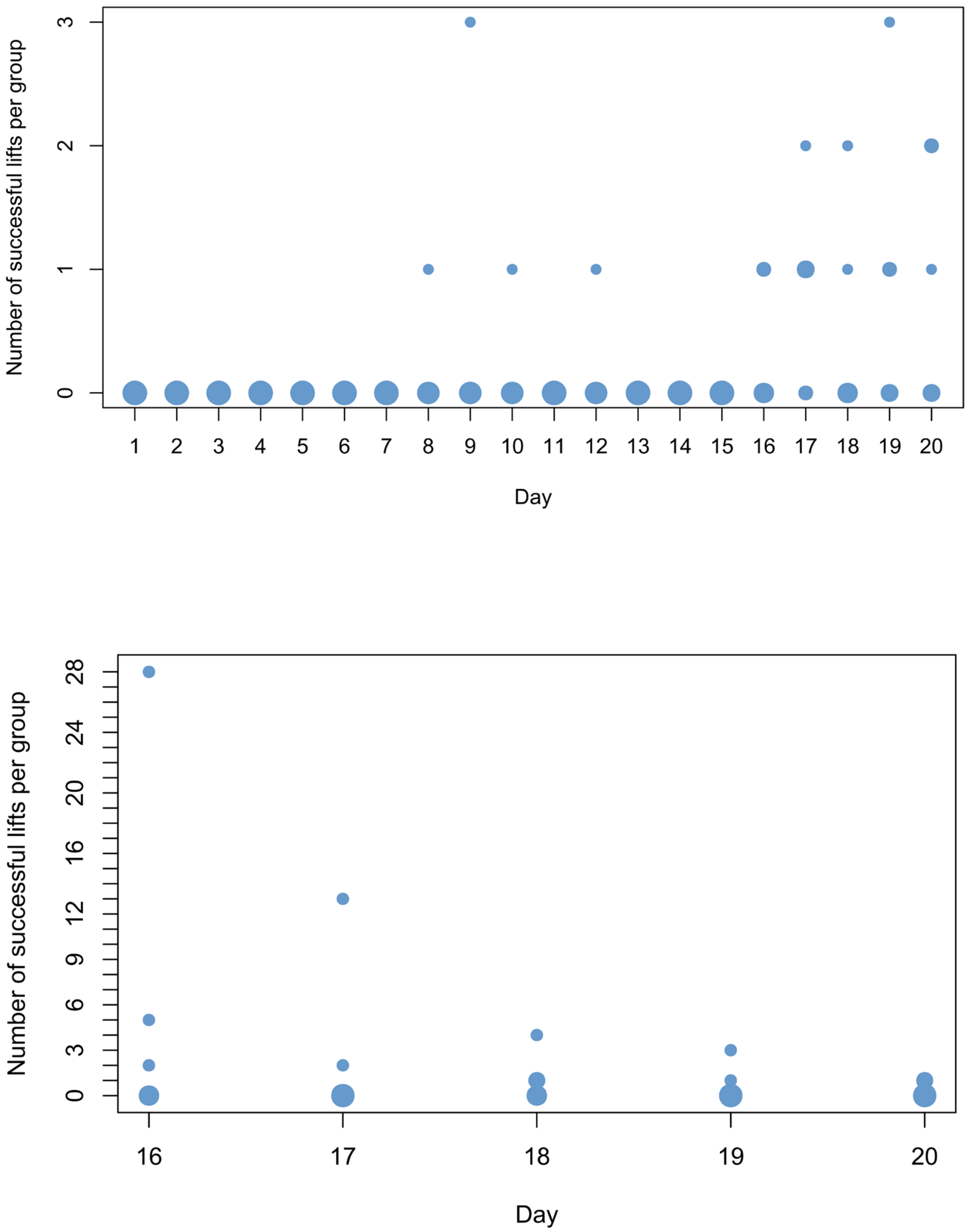

Supplementary Figure 9 Number of successful lifts with the joint log-lift (JLL) box per group each day in the pilot study in the home enclosure (top) and in smaller groups in the test enclosure (bottom). The area of the dots depicts the frequency of the indicated number of successful lifts per day.

9. References

1 Baayen, R. H. *Analyzing linguistic data: A practical introduction to statistics using R*. (Cambridge University Press, 2008).

2 Schielzeth, H. & Forstmeier, W. Conclusions beyond support: overconfident estimates in mixed models. *Behavioral ecology* **20**, 416-420, doi:10.1093/beheco/arn145 (2008).

3 Barr, D. J., Levy, R., Scheepers, C. & Tily, H. J. Random effects structure for confirmatory hypothesis testing: Keep it maximal. *Journal of memory and language* **68**, 255-278, doi:10.1016/j.jml.2012.11.001 (2013).

4 Field, A. *Discovering statistics using SPSS*. 2nd edn, (Sage Publishing, 2005).

5 Fox, J. & Weisberg, S. *An {R} Companion to Applied Regression*. 3rd edn, (Sage Publishing, 2019).

6 Harrison, X. A. *et al.* A brief introduction to mixed effects modelling and multi-model inference in ecology. *PeerJ* **6**, e4794-e4794, doi:10.7717/peerj.4794 (2018).

7 Forstmeier, W. & Schielzeth, H. Cryptic multiple hypotheses testing in linear models: Overestimated effect sizes and the winner's curse. *Behavioral Ecology and Sociobiology* **65**, 47-55, doi:10.1007/s00265-010-1038-5 (2011).

8 Bates, D., Mächler, M., Bolker, B. & Walker, S. Fitting Linear Mixed-Effects Models Using lme4. *Journal of Statistical Software* **67**, 1-48, doi:10.18637/jss.v067.i01 (2015).

9 Dobson, A. J. *An Introduction to Generalized Linear Models*. 2nd edn, (Chapman & Hall/CRC, 2002).

10 McCullagh, P. & Nelder, J. A. *Generalized Linear Models*. 2nd edn, (Chapman & Hall/CRC, 1989).

11 Lenth, R. emmeans: Estimated Marginal Means, aka Least-Squares Means. R package version 1.8.6, https://CRAN.R-project.org/package=emmeans (2023).

12 Rault, J. L., Camerlink, I., Goumon, S., Mundry, R. & Špinka, M. The Joint Log-Lift Task: A Social Foraging Paradigm. *Frontiers in veterinary science* **8**, 745627-745627, doi:10.3389/FVETS.2021.745627 (2021).
